# Null models confirm nest site fidelity by male smallmouth bass, *Micropterus dolomieu*

**DOI:** 10.1186/s40850-024-00205-z

**Published:** 2024-06-27

**Authors:** Daniel D. Wiegmann, Kelly L. Weinersmith, Jeffrey R. Baylis, Steven P. Newman, Lisa M. Angeloni

**Affiliations:** 1 Department of Biological Sciences and J. P. Scott Center for Neuroscience, Mind and Behavior, Bowling Green State University, Bowling Green, OH 43403 USA; 2 Department of BioSciences, Rice University, MS-140, 6100 Main Street, Houston, TX 77005 USA; 3 Department of Integrative Biology, University of Wisconsin, Madison, WI 53706 USA; 4 Wisconsin Department of Natural Resources, Woodruff, WI 54568 USA; 5 Department of Biology, Colorado State University, Fort Collins, CO 80523 USA

**Keywords:** Nest site fidelity, Null models, Smallmouth bass

## Abstract

**Background:**

Many animals appear to preferentially renest in proximity to a site they previously occupied. Evidence of nest fidelity is often inferred from a right skewed distribution of distances between the nests of individuals that breed in two consecutive reproduction episodes, where many individuals nest some arbitrarily close distance to their prior nest and others, in the extended right tail of the distribution, nest far from the nest they previously occupied. Because right skewed distributions of inter-nest distances can arise even when individuals choose nest locations randomly, however, such inferences are prone to error. The importance of null models—used to generate patterns of individual inter-nest distances by processes that do not involve site attachment—for inferences about site fidelity has been known for decades but is still often unappreciated or ignored.

**Methods:**

The right skewed distributions of inter-nest distances observed in two earlier studies of male smallmouth bass (*Micropterus dolomieu*) suggest *prima facie* that males exhibit nest site fidelity between annual reproduction episodes, but patterns of inter-nest distances have yet to be compared to an adequate null model. Here, we evaluate the nest site fidelity of marked male *M*. *dolomieu* in a decade-long dataset, where we apply a randomization procedure based on the *rencontre* probability problem to generate null models. Eight observed distributions of individual, annual inter-nest distances are compared to a year-specific null model to determine whether random processes are sufficient to explain the observed distributions of inter-nest distances.

**Results:**

Through contrasts between observed annual inter-nest distances and results derived from null models that imposed realistic constraints on behavior, we show that some males were undoubtedly nest-site faithful. To reinforce the utility of null models and to make these kinds of models more accessible, we also provide a supplemental tutorial. The tutorial illustrates how random site choices, subject to common ecological and behavioral constraints, and even how distance is measured, can produce patterns of inter-nest distances that falsely imply nest site fidelity, or a lack of fidelity. The R code needed to reproduce these null models is included. The inference errors evident in our examples generalize to other forms of site fidelity, such as the apparent patch fidelity of certain sea bird foragers.

**Conclusions:**

The comparisons of observed distributions of inter-nest distances with those generated by null models imply that, as suggested in prior studies, male *M*. *dolomieu* indeed exhibit annual nest site fidelity. Procedures like those we apply are necessary first steps in analyses when distributions of distances between the nests of individuals in consecutive reproduction episodes are used to infer nest-site fidelity. Why male *M. dolomieu* are site faithful is a question yet to be answered.

**Supplementary Information:**

The online version contains supplementary material available at 10.1186/s40850-024-00205-z.

## Background

Ecologists regularly study spatial patterns to infer ecological processes [1]. Point patterns—mapped point locations—are often the focus of analyses and numerous methods have been developed to identify the processes associated with diverse patterns [2–4]. For example, behavioral ecologists infer processes, like site fidelity, from point patterns. The paired nest locations of individuals that breed in two consecutive reproduction episodes—say, twice in a season or in two successive annual seasons—are expected to be near one another when individuals are site faithful. Indeed, the site fidelity of some birds is truly extraordinary, where individuals may travel thousands of kilometers from their nest sites in spring to an over-winter site and then return in the subsequent spring to breed in nearly the exact same locations [5–7]. Point patterns likewise suggest that nest site fidelity is common in mammals, reptiles, amphibians and fishes, perhaps due in part to benefits derived from a familiarity with a particular site [7].

**Fig. 1:**
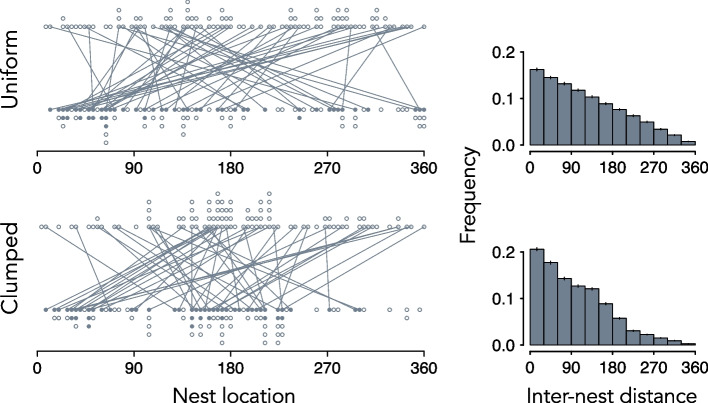
Relationship between the distribution of suitable nest habitat and inter-nest distances when individuals choose nest locations randomly on a hypothetical, linear shoreline of length 360 arbitrary units in two reproduction episodes. Dot plots show 100 randomly chosen points—nest locations—in the interval [0,, 360] when (*top*) suitable nest habitat is uniformly distributed or (*bottom*) clumped, with 50 of the 100 sites in the interval [135, 225]. The upper and lower rows represent the sites occupied in Episode 1 and Episode 2, respectively, and solid dots in Episode 2 show the randomly chosen nest locations of 50 individuals that also bred in Episode 1. Lines project back to their nest locations in Episode 1. Histograms summarize the expected frequencies ($\overline{X}\pm 2 \text{SE })$ of inter-nest distances, based on 1,000 simulations, between paired nest locations of individuals that bred in both Episodes 1 and 2

Nest site fidelity is often inferred from a right skewed distribution of distances between the consecutive nests of an individual, where a preponderance of individuals nest at or near their former site and others, in the long right tail of the distribution, renest far from the site they previously occupied. However, random processes, unrelated to site attachment, can generate movement patterns that are seemingly biased toward previously visited or occupied locations. For example, imagine a situation in which nests are built on a straight section of an ocean beach or a linear stretch of river shoreline, where individuals choose nest locations on the same section of beach or shoreline randomly in each of two consecutive reproduction episodes. In this simple scenario, the distribution of distances between the first and second nest for individuals that breed in both episodes is expected to be strongly right skewed, a pattern that falsely implies some degree of site faithfulness (Fig. 1). This example illustrates why comparisons of point patterns to those generated by null models—the anticipated occupancy of sites based on a random choice of locations, subject to relevant ecological or behavioral constraints—are essential for inferences related to site fidelity [3, 8–11]. Their use in studies of nest site fidelity is, however, still regularly neglected or overlooked, as others have noted [10, 12, 13]. In a Supplemental File, we elaborate on the utility of null models and illustrate how common ecological and behavioral constraints—and even how distance is measured—can impact the expected pattern of distances between the nests of individuals that breed in two consecutive reproduction episodes when nest sites are randomly selected.

Two earlier studies documented inter-annual nest site locations of individual male smallmouth bass (*Micropterus dolomieu*). In both studies, there appears to be an overabundance of close inter-nest distances, a pattern that superficially suggests some level of male nest site fidelity [14, 15, 16]. Here, and in our Supplemental File, we build null models that are based on a well-known probability problem, with some added behavioral constraints, for comparison to the actual inter-nest distances observed in a long-term study on male *M*. *dolomieu* to show that at least some males in the study were nest site faithful.

## Methods

The data we analyzed were collected as part of a long-term study of *M*. *dolomieu* reproductive behavior conducted in 1999, 2001–2009. Because our interest was in nest site fidelity and *M. dolomieu* spawn in a discrete season, once per year, our analyses were confined to the contiguous years of the study, 2001–2009. The field methods we used follow those described elsewhere [17–20]. Here, we briefly reiterate those methods that are specifically relevant to our analyses.

### Study species

Reproduction by *M. dolomieu* in northern populations begins when water temperatures approach 15 ºC [21, 22]. Larger males establish a nest site and spawn earlier in a season than smaller males [18, 19, 23–25]. Typically, males mate monogamously [16–18, 26]. Parental care is paternal and males remain at the nest and defend progeny until they swim up and disperse, a period that may last several weeks [27–29].

Nests are built in the littoral zone and, while male *M. dolomieu* are territorial, solitary nesters, the distribution of suitable habitat may result in aggregations of nests [30, 31]. Because of water level fluctuations, allochthonous inputs, or anthropogenic disturbances in the nearshore littoral zones of lakes, however, what constitutes a good location for a nest probably varies between years [32].

### Study site

**Fig. 2:**
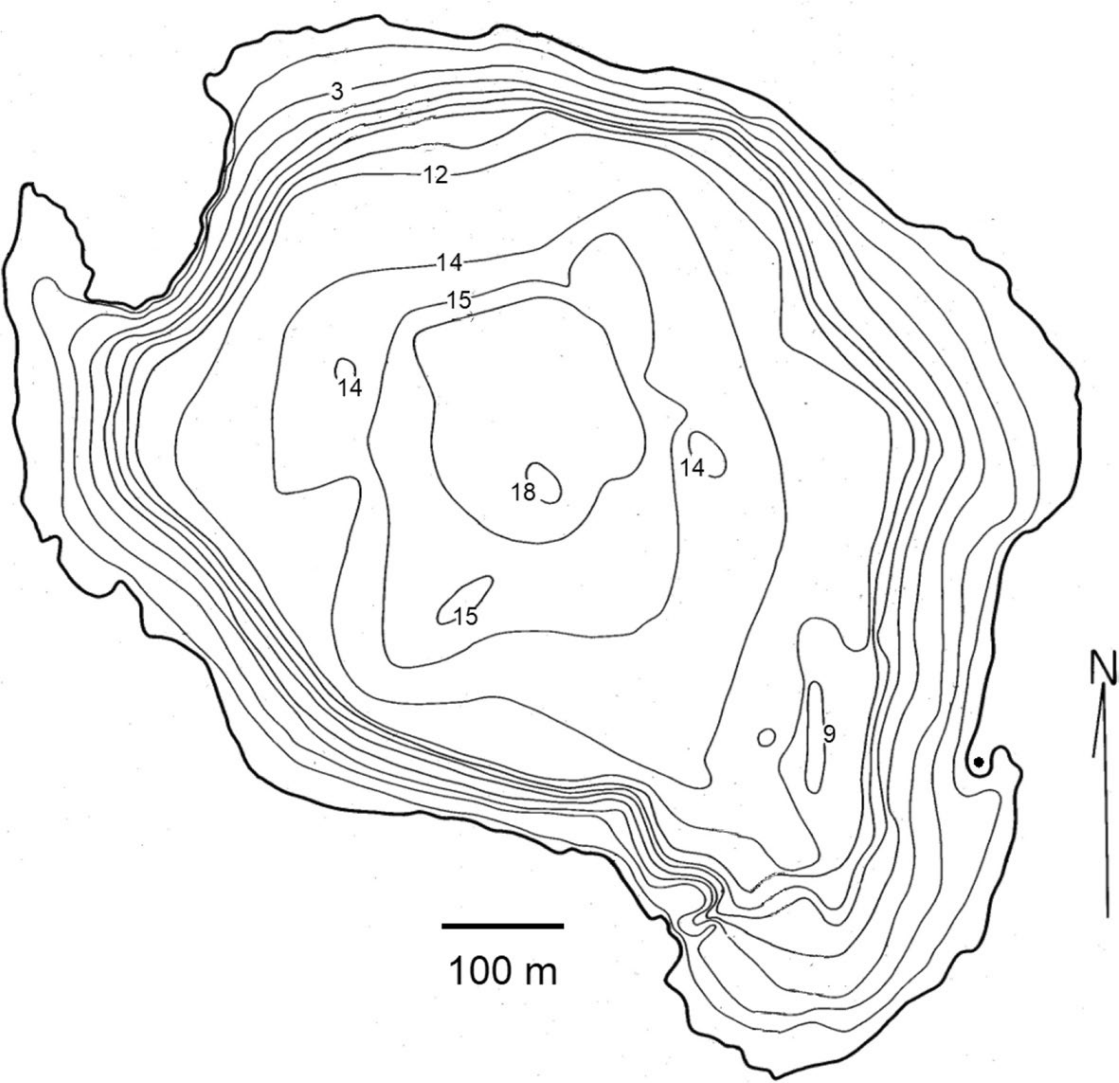
Bathymetric map of Pallette Lake that shows contours in depth increments of approximately 1.5 m. Male smallmouth bass (*Micropterus dolomieu*) nests are typically built at depths of 1–3 m. The solid dot on the eastern shoreline shows the approximate position of the stationary GPS unit used to map nest locations. [Adapted from Wisconsin Department of Natural Resources public image: https://dnr.wi.gov/lakes/maps/DNR/1872100a.pdf.]

The study was conducted on Pallette Lake, a roughly circular 70-ha seepage lake located in north-central Wisconsin (46.067 N, 89.604 W). The littoral zone of the lake is largely sand, with stretches of gravel, cobble, and rubble [33]. The lake has a maximum depth of 18 m and a shoreline of about 4 km (Fig. 2). Importantly, the system is closed so that *M. dolomieu* cannot migrate and spawn elsewhere [17].

### Nest census

Pallette Lake was surveyed daily by snorkelers and observers in boats from mid-May through June and, if necessary, early July to locate active nests—nests with eggs or hatched larvae—guarded by parental males. Each nest was marked with a uniquely numbered waterproof paper tag attached to a sinker, positioned near the nest perimeter. When a season ended, paper tags were replaced with permanent markers, if one was not already present, which allowed us to subsequently ascertain the exact location of previously used nests.

### Identification of parental males

In each year, we attempted to capture males from all active nests. Parental males were captured with a hand net, marked with a uniquely numbered Floy FD-67C anchor tag for future identification, if they were not already tagged, and were promptly released. Wiegmann and Baylis [20] describe the capture procedures we used in detail.

### Nest locations

Two Magellan™ GPS ProMARK X™ receivers were used to collect GPS coordinates for active nests, where one unit was positioned as a base station on the shoreline at the same location each year and the other was held over nests (Fig. 2). The shoreline receiver ran continuously and its static position allowed us to use differential GPS to adjust real-time GPS signals and thereby reduce pseudo-range errors. The hand-held receiver was positioned over a nest until a minimum of 120 points were collected, which were then averaged to further improve positional accuracy. Both receivers were set to collect data once per second. The precision of GPS data based on computed straight-line distances between nests located within 1 m of the same permanent marker in two consecutive years was $\overline{X}\pm 1\text{SE }=3.54\pm 0.07$ m (*N* = 911).

These GPS coordinates were used to compute the straight-line distance between nests of males identified by Floy tag numbers to have spawned in two consecutive years, hereinafter referred to as *repeat breeders*. Occasionally, males spawned more than once in a season and were captured from two, or in rare instances, three nests. In these instances, the observed annual inter-nest distance was computed from the last nest occupied in the first year to the first nest occupied in the second year.

### Null models

The approach we used to analyze our field data was inspired by what is known as the *rencontre* probability problem [34]. The basic idea is to determine the probability that some specified number of objects in a set would be matched correctly by chance to objects in another set to which they are paired. For instance, if seven individuals that constructed nests and bred in one season returned to breed in a second season and all seven previously built nests—and only those seven nests—were available, *rencontre* numbers allow us to determine the probability that, say, four or more individuals would renest by chance at the site they previously occupied. (The answer is approximately 0.0183.)

Pledger and Bullen [8] modified the *rencontre* problem to verify the supposed mate and nest site fidelity of blue penguins, *Eudyptula minor *[12]. There are two notable aspects to their approach as applied to nest-site fidelity: they used the locations of nests built in the second season as candidate choices; and they modified the problem to allow repeat breeders to choose from all nests built in the second season, rather than just the available nests used by repeat breeders in the previous year. Together, their modifications simultaneously specify the location of suitable nest habitat in the second reproduction episode and control for behavior, like territoriality, that might impact how nests in the second season are spaced. Their approach is, however, overly restrictive because repeat breeders are declared to be site faithful only if the same *exact* nest location is used in both reproduction episodes. The essence of their method can nonetheless be maintained when this restriction is relaxed, as in our approach, and the *distances* between nests of individual repeat breeders, rather than the number of site *matches*, is used to evaluate site faithfulness.

Null models were generated with R, Version 4.0.5 [35]. In these models, repeat breeders that spawned in years *t* and *t* + 1 and *new* breeders—males that were untagged when they were captured—that spawned in year *t* + 1 were assigned randomly to nests in which eggs were deposited in year *t* + 1. The straight-line distance between the actual nest locations of repeat breeders in year *t* and their randomly assigned locations in year *t* + 1 was then computed. This process was repeated 1,500 times for each pair of consecutive years to generate null distributions against which we could compare the observed distribution of inter-year nest distances of repeat breeders. This *rencontre*-based procedure imposed constraints on the location of suitable habitat and on allowable distances between nests due to territorial behavior. These implicit constraints were the only constraints imposed in our basic null model. In the Supplemental File, we incorporate additional behavioral constraints—earlier reproduction by larger males, typically repeat breeders, and habitat preferences—into our basic null model. (Null models with these added constraints yield the same conclusion as our basic model with regard to male *M. dolomieu* nest site faithfulness.) No movement constraints were imposed in null models because the maximum distance between two nests in Pallette Lake is about 1,200 m and males in some populations are known to migrate 10,000 m from over-winter sites to spawn (Fig. 2; [36]).

### Analyses

Null models are expected to generate distributions of inter-nest distances for repeat breeders that are similar to those we observed if no male *M. dolomieu* were site faithful. Two approaches were used to determine whether distributions matched. First, we compared the observed distribution to each of the 1,500 null model distributions by use of two-sample Kolmogorov–Smirnov *D* statistics, which are simultaneously sensitive to differences in centrality and the shape of distributions [37]. Because our analyses involved a large number of comparisons and *D* is sensitive to sample sizes and, in some years, the number of repeat breeders in our data set was relatively large, we used a significance level of $\alpha$ = 0.001.

Second, we evaluated whether specific parameters related to the centrality or dispersion of the observed and null distributions of inter-nest distances differed. For each observed distribution, we computed its mean, median, variance, skew and kurtosis. These statistics were likewise computed for each of the 1,500 null distributions for each pair of years to generate a distribution for each statistic for each year pair. The value of an actual, observed statistic should be contained within the simulated distribution for that statistic if the simulation process replicates the realized behavior. The equivalent of a probability value—denoted herein as Ω—for each observed statistic was computed, where the reference distribution was the distribution of the respective statistic generated by the 1,500 simulations [11]. Here, we concluded that the random assignment of males to nests under a null model replicated an observed statistic when Ω > 0.05. The observed proportions of males that occupied the same nest in two consecutive years were likewise compared to the respective proportions generated by null models.

## Results

### Field data

Females spawned in 170 to 329 nests in 2001–2009 and parental males were captured from 89 to 98 percent of these nests ($\overline{X }\pm 1 SE=0.94\pm 0.01$; Table 1). Nearest-neighbor distances averaged between 10 m ($\overline{X }\pm 1 SE=10.4\pm 0.4$ m) in 2004, the year with the highest density of nests, and 15 m ($\overline{X }\pm 1 SE=15.3\pm 0.8$ m) in 2007, the year with the lowest nest density, with an overall average of about 12 m ($\overline{X }\pm 1 SE=12.0\pm 0.2$ m). The number of unique males captured from nests in a season ranged from 152 to 284. As many as 14 percent ($\overline{X }\pm 1 SE=0.06\pm 0.02$) of these males spawned two or, occasionally, three times in the same year. The distance between the nests of these males, if they returned to breed the next year, was computed, as noted earlier, from the location of the last nest they occupied in the first year to the location of the first nest they occupied in the second year.

**Table 1:** Descriptive data for parental male smallmouth bass (*Micropterus dolomieu*) in Pallette Lake (Wisconsin, USA) from 2001–2009. *Nests* indicates the total number of nests in which fertilized eggs were found. *Unique Males* is the *Total* number of unique males captured from nests; *Number of Nests* indicates how many of these males spawned multiple times and were captured from 1, 2 or 3 nests in a year.* *Repeat Breeders* provides similar information on the subset of unique males that bred in the next year

		Unique Males^a^				Repeat Breeders			
			Number of Nests				Number of Nests		
Year	Nests^a^	Total	1	2	3	Total	1	2	3
2001	171	152	140	12	0	79	73	6	0
2002	241	221	211	10	0	142	135	7	0
2003	268	234	209	23	2	146	128	17	1
2004	329	284	245	39	0	159	137	22	0
2005	242	216	216	0	0	127	127	0	0
2006	214	188	185	3	0	116	115	1	0
2007	170	157	155	2	0	108	106	2	0
2008	211	198	195	2	1	133	131	1	1
2009	215	176	154	22	0				

^a^The numbers under the 1, 2 and 3 headers when summed equal the *Total*. In 2001, for instance, 140 of the 152 total unique males were each captured from one nest, 12 males spawned twice and were captured from two nests and no males spawned three times so the total number of captures was 140(1)+12(2)+0(3)=164

**Fig. 3:**
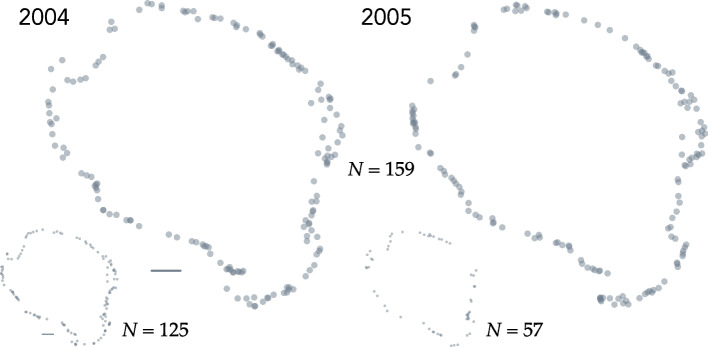
Nest locations of the 159 smallmouth bass (*Micropterus dolomieu*) males known to have spawned in both 2004 and 2005 in Pallette Lake (Wisconsin, USA). The smaller plots (lower left) show the nest locations of the 125 males captured in 2004 but not in 2005 and (lower right) the nest positions of the 57 captured new breeders in 2005. Line segments are 100 m

**Fig. 4:**
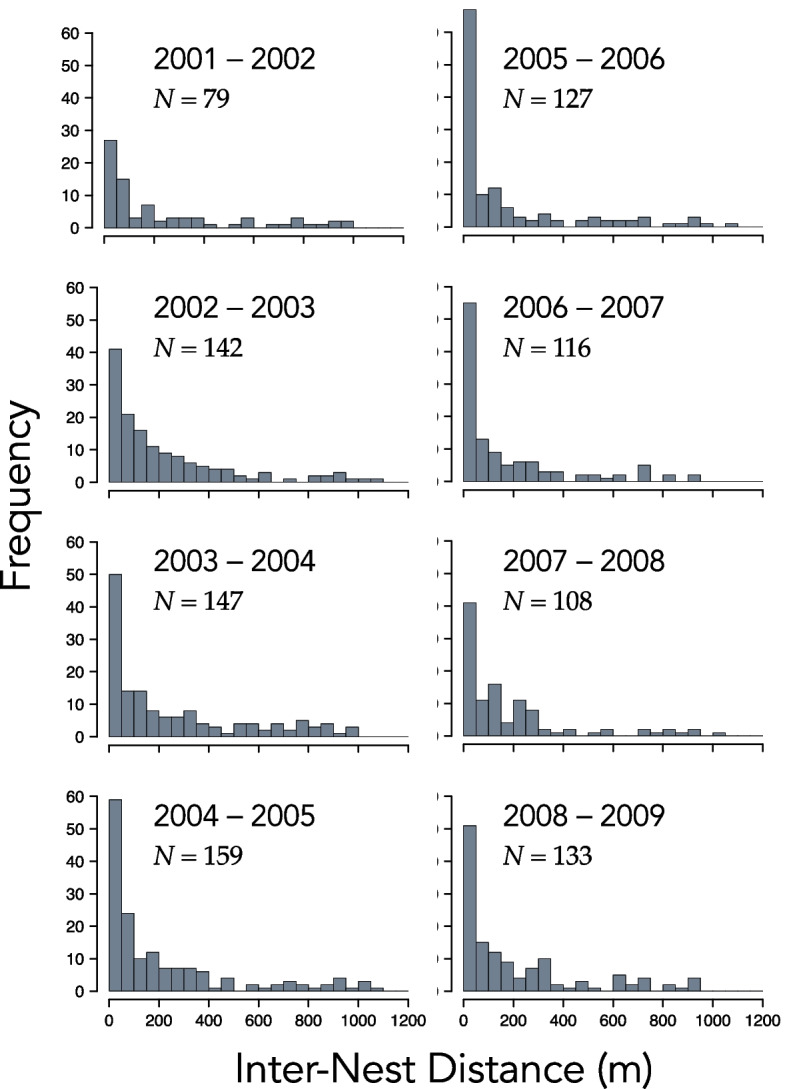
Histograms of the distance between nests of individual male smallmouth bass (*Micropterus dolomieu*) that bred in consecutive years in Pallette Lake (Wisconsin, USA)

Between 52 and 68 percent ($\overline{X }\pm 1 SE=0.61\pm 0.02$) of the males captured in 2001–2008 were captured again in the subsequent year. Figure 3 shows the nest locations of males that bred in 2004, the year with the highest density of nests, and again in 2005. The distribution of distances between the nests of the 79 to 159 repeat breeders was strongly right skewed in all eight pairs of consecutive years of the study (Fig. 4). The median inter-nest distance amongst repeat breeders ranged from approximately 46 m to 120 m (Table 2). The mean was considerably larger—172 m to 260 m—as expected based on the right skew of inter-nest distances. The proportion of repeat breeders that guarded the exact same nest in two consecutive years ranged between 0.06 and 0.18 ($\overline{X }\pm 1 SE=0.12\pm 0.01$). But some males renested a long distance from the location of the nest they occupied the year earlier. The maximum observed inter-nest distance ranged between 937 and 1090 m ($\overline{X }\pm 1 SE=1008\pm 21.59$ m).

**Table 2:** Inter-nest distance summary statistics. The observed (*O*) values of statistics and expected (*E*) values based on 1,500 simulated inter-nest distance distributions under our base null model. Bolded values of expected variances indicate that the observed variance was contained in the distribution of the 1,500 simulated variances (Ω>0.05). *Proportion* indicates the frequency of repeat breeders that spawned at the same location in consecutive years

	Statistics											
	Mean		Median		Variance		Skew		Kurtosis		Proportion	
Episodes	*O*	*E*	*O*	*E*	*O*	*E*	*O*	*E*	*O*	*E*	*O*	*E*
2001–2002	231	586	88	629	82,909	**89,927**	1.33	-0.27	3.47	1.92	0.1519	0.0010
2002–2003	219	582	118	621	65,061	92,072	1.63	-0.23	5.02	1.87	0.1056	0.0004
2003–2004	255	575	116	614	81,765	**87,192**	1.09	-0.22	2.90	1.92	0.0890	0.0003
2004–2005	219	583	97	631	78,009	**86,231**	1.60	-0.27	4.54	1.96	0.0566	0.0003
2005–2006	178	590	46	640	70,365	87,159	1.76	-0.30	5.02	1.90	0.1811	0.0008
2006–2007	172	582	60	630	54,090	85,203	1.72	-0.28	5.07	1.99	0.0948	0.0005
2007–2008	183	561	108	599	56,168	84,329	1.89	-0.22	5.93	1.93	0.1296	0.0016
2008–2009	208	561	103	604	64,668	83,027	1.45	-0.26	4.11	1.91	0.1400	0.0015

### Field data comparison to null models

**Fig. 5:**
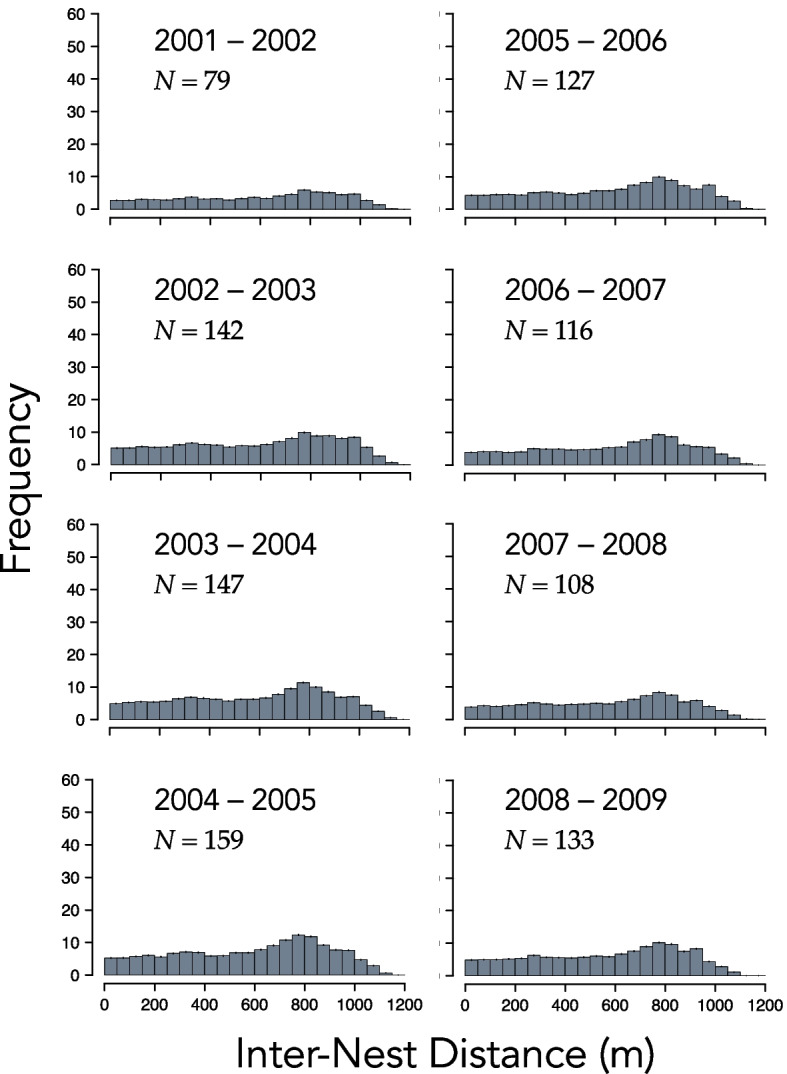
Expected frequencies (±2 SE) of inter-nest distances between the nests of male smallmouth bass (*Micropeterus dolomieu*) that bred in consecutive years in Pallette Lake (Wisconsin, USA) based on 1,500 simulations under the basic null model

The distributions of distances between the successive nests of repeat breeders generated by our basic null model were much flatter than the observed distributions (Fig. 5). Indeed, none of the 1,500 null distributions generated for each of the eight pairs of consecutive seasons matched their respective observed distribution of inter-nest distances: every two-sample Kolmogorov–Smirnov test resulted in a rejection of the null hypothesis that the simulated and observed distributions are identical at a significance level of $\alpha$ = 0.001.

The simulations also failed to replicate the measured parameters of observed inter-nest distance distributions of repeat breeders except for the variance of distances between 2001 and 2002 (Ω = 0.4353), 2003 and 2004 (Ω = 0.4100) and 2004 and 2005 (Ω = 0.1833) (Table 2). The observed values of the other four distribution statistics—mean, median, skew and kurtosis—were not contained in simulated distributions (Ω < 0.0007). Hence, the null models failed to replicate any of these parameters. Notably, the simulated mean and median inter-nest distance were much larger than the observed values in all years, which implies that overall males nested closer to their previous nests than expected under the null models. In particular, the difference between mean inter-nest distance generated by null models and observed means ranged between 320 and 412 m ($\overline{X }\pm 1 SE=369 \pm 11$ m). The difference between null model medians and observed medians was even larger ($\overline{X }\pm 1 SE=594 \pm 13$ m). In null model simulations, repeat breeders were also rarely assigned to the nest they occupied the year earlier. The mean proportion of males assigned by null models to the nest they occupied the previous year ranged from 0.0003 to 0.0016 ($\overline{X }\pm 1 SE=0.0008 \pm 0.00018$; Table 2). Indeed, none of the observed proportions were contained in simulated distributions (Ω < 0.0007).

## Discussion

The right-skewed distributions of distances between the nests of individual male *M. dolomieu* between seasons in two earlier studies suggested that male *M. dolomieu* are site faithful [14, 15]. Ridgway et al. [14] recognized the need for a null model and, for lack of a better theoretical alternative, used a uniform distribution as a reference for comparison to the distribution of inter-nest distances they observed. Unfortunately, there is probably no well-defined distribution that might serve as a general null model. Instead, a spatially explicit map of suitable nest habitat and some knowledge of the mobility, behavior, and habitat preferences of the study organism are needed for the construction of null models. In the Supplemental File, we provide R code to facilitate the formulation of null models. To reinforce the utility of null models, we also construct some simple examples that clearly illustrate how nest choices that involve no site attachment can generate distributions of inter-nest distances that are right skewed, deceitful indicators of site fidelity, or left-skewed, patterns speciously reflective of anti-philopatry. The examples also reveal why null models are useful when nest site fidelity is inferred from other spatial patterns, situations in which null models might seem unnecessary, as when the distances between successive nests of winners are compared to those of losers under a win-stay, lose-shift strategy [38–41].

In our study of *M. dolomieu*, the distribution of distances between the nests of repeat breeders in each of eight pairs of consecutive years was strongly right skewed. The *recontre*-based null models we formulated, which implicitly accounted for the location of suitable habitat and behavioral interactions that influenced how nests were spaced, failed to replicate any of the distributions we observed. Notably, the observed distances between the nests of repeat breeders were in all cases much closer than those simulated under the basic null model. The observed proportion of males that renested in the same location in consecutive years was also far larger than those simulated. Null models with added behavioral constraints, included in our Supplemental File, performed no better than the basic null model. Together, these results provide strong evidence that at least some males in our study population were site faithful. Indeed, the stark contrast between observed inter-nest distances and those generated by null models suggests that many males may exhibit some level of site fidelity (Figs. 4 and 5).

Why male *M. dolomieu* are site faithful is yet to be determined, but site fidelity is generally suspected to confer fitness benefits [7]. Perhaps more mysterious are individuals that renest long distances from the nests they previously occupied, individuals in the right tail of inter-nest distance plots (Fig. 4). In each pair of years of our study, the straight-line distance between nests of some repeat breeders exceeded 900 m and in four of the eight pairs of years was farther than 1,000 m, or roughly 1,000–1,150 m of shoreline (Figs. 2 and 4). Recent studies have documented an increase in the distance between nests of male *M. dolomieu* and male largemouth bass (*Micropterus nigricans*) repeat breeders in response to anthropogenic disturbances [42, 43]. Detailed studies of the causes and fitness consequences of long-distance shifts of nest locations by repeat breeders would provide insights into both the benefits of nest site fidelity and the extent to which we should be concerned about anthropogenic disturbances on nest site choices.

## Conclusions

The distribution of distances between the nests of individuals that breed in two consecutive reproduction episodes can falsely imply either nest site fidelity or a lack of site fidelity. Hence, an evaluation of site fidelity based on patterns of site reuse is strengthened by a comparison to a null model. Pledger and Bullen [8] devised a useful method to generate null models of site fidelity based on the *rencontre* probability problem that simultaneously controls for unmeasured factors that determine the location of suitable nest habitat and any interactions amongst individuals, like territoriality, that may influence where nests are constructed. The procedure imposes an overly restrictive definition of site fidelity, which can be relaxed to devise null models that incorporate additional relevant constraints on behavior, such as limited movement or habitat preferences. Then, well-known procedures can be used to contrast observed inter-nest distance distributions with those generated by null models [11]. Through such contrasts between observed inter-nest distances and results derived from null models that imposed realistic constraints on behavior, we provide evidence that some—perhaps many—male *M. dolomieu* repeat breeders in our study population were nest-site faithful, as appears to occur in other *M. dolomieu* populations.

### Supplementary Information

Supplementary Material 1.

Supplementary Material 2.

Supplementary Material 3.

Supplementary Material 4.

Supplementary Material 5.

Supplementary Material 6.

Supplementary Material 7.

Supplementary Material 8.

Supplementary Material 9.

Supplementary Material 10.

Supplementary Material 11.

Supplementary Material 12.

## Data Availability

The dataset used in the current study is available, on reasonable request, from the corresponding author.

## References

[1] Fletcher, R, Fortin M-J. Spatial Ecology and Conservation Modeling: Applications with R. Switzerland: Springer Nature Switzerland AG; 2018.

[2] Perry JN, Liebhold AM, Rosenberg MS, Dungan J, Miriti M, Jakomulska A, Citron-Pousty S (2002). Illustrations and guidelines for selecting statistical methods for quantifying spatial pattern in ecological data. Ecography.

[3] Wiegand T, Moloney KA (2004). Rings, circles, and null-models for point pattern analysis in ecology. Oikos.

[4] Velázquez E, Martínez I, Getzin S, Moloney KA, Wiegand T (2016). An evaluation of the state of spatial point pattern analysis in ecology. Ecography.

[5] Greenwood PJ (1980). Mating systems, philopatry and dispersal in birds and mammals. Anim Behav.

[6] Greenwood PJ, Harvey PH (1982). The natal and breeding dispersal of birds. Annu Rev Ecol Syst.

[7] Piper WH (2011). Making habitat selection more “familiar”: a review. Behav Ecol Sociobiol.

[8] Pledger S, Bullen L (1998). Tests for Mate and Nest Fidelity in Birds with Application to Little Blue Penguins (*Eudyptula minor*). Biometrics.

[9] Gotelli NJ, Graves GR (1996). Null Models in Ecology.

[10] Richardson TO, Giuggioli L, Franks NR, Sendova-Franks AB (2017). Measuring site fidelity and spatial segregation within animal societies. Methods Ecol Evol.

[11] Cranmer K, Brehmera J, Louppec G (2020). The frontier of simulation-based inference. Procedings of the National Academy of Sciences.

[12] Schaefer JA, Bergman CM, Luttich SN (2000). Site fidelity of female caribou at multiple spatial scales. Landscape Ecol.

[13] Campbell SP, Witham JW, Hunter ML (2010). Stochasticity as an alternative to deterministic explanations for patterns of habitat use by birds. Ecol Monogr.

[14] Ridgway MS, MacLean JA, MacLeod JC (1991). Nest-site fidelity in a centrarchid fish, the smallmouth bass (*Micropterus dolomieui*). Can J Zool.

[15] Barthel BL, Cooke SJ, Svec JH, Suski CD, Bunt CM, Phelan FJS, Philipp DP (2008). Divergent life histories among smallmouth bass *Micropterus dolomieu* inhabiting a connected river–lake system. J Fish Biol.

[16] Franckowiak RP, Ridgway MS, Wilson CC (2017). Genetic mating system and mate selection in smallmouth bass. Ecol Evol.

[17] Raffetto NS, Baylis JR, Serns SL (1990). Complete estimates of reproductive success in a closed population of smallmouth bass (*Micropterus dolomieui*). Ecology.

[18] Wiegmann DD, Baylis JR, Hoff MH (1992). Sexual selection and fitness variation in a population of smallmouth bass, *Micropterus dolomieui* (Pisces: Centrarchidae). Evolution.

[19] Wiegmann DD, Baylis JR, Hoff MH (1997). Male fitness, body size and timing of reproduction in smallmouth bass, *Micropterus dolomieui*. Ecology.

[20] Wiegmann DD, Baylis JR (1995). Male body size and paternal behaviour in smallmouth bass, *Micropterus dolomieui* (Pisces: Centrarchidae). Anim Behav.

[21] Hubbs C. L. and R. M. Bailey. 1938. The small-mouthed bass. Cranbrook Institute of Science Bulletin 10. 92 pp.

[22] Shuter BJ, Maclean JA, Fry FEJ, Regier HA (1980). Stochastic Simulation of Temperature Effects on First-Year Survival of Smallmouth Bass. Trans Am Fish Soc.

[23] Ridgway MS, Shuter BJ, Post EE (1991). The relative influence of body size and territorial behaviour on nesting asynchrony in male smallmouth bass, *Micropterus dolomieui* (Pisces: Centrarchidae). J Anim Ecol.

[24] Baylis JR, Wiegmann DD, Hoff MH (1993). Alternating life histories of smallmouth bass. Trans Am Fish Soc.

[25] LaRoche RAS, Weinersmith KL, Davis ML, Angeloni LA, Baylis JR, Newman SP, Egan SP, Wiegmann DD (2023). Size-associated energetic constraints on the seasonal onset of reproduction in a species with indeterminate growth. Oikos.

[26] Ridgway MS, Goff GP, Keenleyside MHA (1989). Courtship and spawning behavior in smallmouth bass (*Micropterus dolomieui*). Am Midl Nat.

[27] Gross ML, Kapuscinski AR (1997). Reproductive success of smallmouth bass estimated and evaluated from family- specific DNA fingerprints. Ecology.

[28] Hinch SG, Collins NC (1991). Importance of diurnal and nocturnal nest defense in the energy budget of male smallmouth bass: insights from direct video observations. Trans Am Fish Soc.

[29] Ridgway, M. S., B. J. Shuter, T. A. Middel and M. L. Gross. 2002. Spatial ecology and density-dependent growth in smallmouth bass: the juvenile transition hypothesis. In Black bass: ecology, conservation and management: 47–60. Philipp, D.P. and M. S. Ridgway, M.S. (Eds). Bethesda, MD. American Fisheries Society.

[30] Rejwan C, Shuter BJ, Ridgway MS, Collins NC (1997). Spatial and temporal distributions of smallmouth bass (*Micropterus dolomieu*) nests in Lake Opeongo, Ontario. Canadian Journal of Aquatic Sciences.

[31] Miller AD, Brewer SK (2021). Riverscape nesting dynamics of Neosho smallmouth bass: to cluster or not to cluster?. Biodiversity and Distributions.

[32] Bozek MA, Short PH, Edwards CJ, Jennings MJ, Newman SP (2002). Habitat selection of nesting smallmouth bass *Micropterus dolomieu* in two north temperate lakes. Am Fish Soc Symp.

[33] Saunders R, Bozek MA, Edwards CJ, Jennings MJ, Newman SP (2002). Habitat features affecting smallmouth bass *Micropterus dolomieu* nesting success in four northern Wisconsin lakes. Am Fish Soc Symp.

[34] Riordan J (1958). An Introduction to Combinatorial Analysis.

[35] R Core Team. 2024. R: A language and environment for statistical computing. R Foundation for Statistical Computing, Vienna, Austria. https://www.R-project.org

[36] Lyons J, Kanehl P (2002). Seasonal movements of smallmouth bass in streams. Am Fish Soc Symp.

[37] Glover T, Mitchell K (2016). An Introduction to Biostatistics.

[38] Robert A, Paiva VH, Bolton M, Jiguet F, Bried J (2014). Nest fidelity is driven by multi-scale information in a long-lived seabird. Proc Biol Sci.

[39] Carroll G, Harcourt R, Pitcher BJ, Slip D, Jonsen I (2018). Recent prey capture experience and dynamic habitat quality mediate short-term foraging site fidelity in a seabird. Proc Biol Sci.

[40] Beal M, Byholm P, Lötberg U, Evans TJ, Shiomi K, Åkesson S (2021). Habitat selection and foraging site fidelity in Caspian Terns (*Hydroprogne caspia*) breeding in the Baltic Sea. Ornis Fennica.

[41] Bonnet-Lebrun A-S, Collet J, Phillips RA (2021). A test of the win-stay—lose-shift foraging strategy and its adaptive value in albatrosses. Anim Behav.

[42] Twardek WM, Shultz AD, Claussen JE, Cooke SJ, Stein JA, Koppelman JB, Phelan FJS, Philipp DP (2017). Potential consequences of angling on nest-site fidelity in largemouth bass. Environ Biol Fishes.

[43] Stegens E, Wiegmann DD, Angeloni LM, Baylis JR, Laroche RAS, Newman SP, Egan SP, Sass GG, Weinersmith KL (2024). Mark-Recapture Surveys Impact Nest Site Fidelity but not Reproductive Timing of Male Smallmouth Bass. North Am J Fish Manag.

