# Supplementary material for: Null models confirm nest site fidelity by male smallmouth bass, Micropterus dolomieu
Source: BMC Zool. 2024 Jun 27;9:13. doi: 10.1186/s40850-024-00205-z (PMC11210175; doi:10.1186/s40850-024-00205-z)
Supplement: Supplementary file 1 — Supplementary Material 1. [file 40850_2024_205_MOESM1_ESM.docx]

**Supplemental Null Model Tutorial**

Daniel D. Wiegmann^1^, Kelly L. Weinersmith^2^, Jeffrey R. Baylis^3^, Steven P. Newman^4^ and Lisa M. Angeloni^5^

^1^Department of Biological Sciences and J. P. Scott Center for Neuroscience, Mind and Behavior, Bowling Green State University, Bowling Green, OH 43402, USA; ^2^Department of BioSciences, Rice University, MS-140, 6100 Main Street, Houston, TX 77005, USA; ^3^Department of Integrative Biology, University of Wisconsin, Madison, WI 53706, USA; ^4^Wisconsin Department of Natural Resources, Woodruff, WI 54568, USA; ^5^Department of Biology, Colorado State University, Fort Collins, CO 80523, USA;

**Correspondence**

Daniel D. Wiegmann, Department of Biological Sciences, Bowling Green State University, Bowling Green, OH 43402; Email: ddwiegm@bgsu.edu; Phone: 419.372.2691

**Background**

Because random processes, unrelated to site attachment, can generate movement patterns that are biased toward previously visited or occupied locations, null models—the anticipated occupancy of sites based on a random choice of locations, subject to ecological and behavioral constraints—are essential for inferences about site fidelity [1; see also 2, 3, 4, 5]. However, the importance of null models for inferences about nest site fidelity, in particular, is still regularly neglected or overlooked, as others have noted [6, 7]. Indeed, site fidelity is often inferred from an apparent preponderance of individuals that nest at or near a site they used in an antecedent reproduction episode—that is, from a right skewed distribution of distances between the nests of individuals that breed in two consecutive reproduction episodes—and is even pseudo-quantified by the proportion of individuals that renest within some *arbitrary* distance from the site they previously occupied. This inadvisable practice has persisted, perhaps, because erroneous conclusions associated with the approach have not been clearly illustrated.

In this supplement, we first illustrate how some common ecological and behavioral constraints—and even how distance is measured—can impact the expected pattern of distances between the nests of individuals that breed in two consecutive reproduction episodes when, within specified constraints, nest sites are randomly selected. The examples we develop elucidate how inference errors can arise when site fidelity is judged solely by patterns of site use and are meant to underpin the utility of null models. The examples are developed in the context of nest site fidelity, but their lessons likewise apply to other forms of site fidelity, such as the site fidelity of foragers [e.g., 8, 9, 10]. In addition, we briefly discuss how the spatial scale over which site fidelity is studied may introduce inference errors. Finally, we further develop the base null model presented in the main paper to illustrate how additional constraints can be added to null models and show that even these more sophisticated null models fail to replicate the distributions of distances between the nests of male *M. dolomieu* observed in our study.

**Formulation of Null Models**

In this supplement, we used R Version 4.0.5 to explore how common ecological and behavioral constraints may influence the expected distribution of distances between the nests of individuals that breed in two consecutive reproduction episodes—hereinafter referred to as *repeat breeders*—under a null model [11; for R code used to generate null models for these examples, see Appendix 1]. The selected examples are not meant to capture the nuanced intricacies of real systems, but instead serve to show how random choices under even simplistic conditions can produce distributions of inter-nest distances and other patterns of site use amongst repeat breeders that *prima facie* imply either the presence or absence of nest site fidelity.

In typical studies of site nest fidelity, the nest locations of marked individuals in an initial episode of reproduction are mapped. Then, in a second episode, which generally also involves new breeders, nests are surveyed to ascertain the positions of repeat breeders, and the distance between the paired points for nests of each repeat breeder is computed. In the scenarios we consider, we suppose that in the first reproduction episode—Episode 1—100 individuals breed and that 50 of these individuals breed again in a second, consecutive episode—Episode 2—when they are joined by 50 new breeders. The 50 repeat breeders are chosen randomly from the 100 breeders in Episode 1 and, in each episode, individuals are assigned to nest locations randomly, subject to any specified ecological or behavioral constraints. For each scenario, we provide an illustration associated with one simulation of the scenario and a histogram, based on 1,000 simulations, that summarizes the expected null distribution of inter-nest distances of repeat breeders for a specified null model.

**Ecological Constraints**

Two ecological constraints are considered: the shape of the landscape and the distribution of nest habitat, which interact to influence the expected null distribution of inter-nest distances of repeat breeders. In particular, we consider a landscape that is linear, circular or distributed in two discrete, linear patches. For each landscape, we suppose that nest habitat is either homogeneous, and nests are uniformly distributed on the landscape, or that a core area supports a higher density of nests, so that nests are spatially clumped.

*Linear landscape*

First, consider a situation in which nests are built on, say, a straight section of an ocean beach or a linear stretch of river shoreline that has a length of 360 arbitrary distance units, where individuals choose nest locations on the same section of beach or shoreline randomly in each of two consecutive reproduction episodes. In this simple scenario, described in the primary paper, the distribution of inter-nest distances of repeat breeders is expected to be strongly right skewed, whether nests are distributed uniformly or clumped (Figure S1). In either situation, the pattern of inter-nest distances falsely implies some degree of site faithfulness.

*Circular landscape*

Next, imagine that nests are constructed around, say, the perimeter of a circular lake (Figure S2). For this example, we simply wrapped the linear data used to generate Figure S1 end-to-end to create a circle with a circumference of 360 arbitrary distance units. If we compute inter-nest distances as the chord between the same pairs of points in Figure S1, there now appears to be an overabundance of long between-episode nest distances, the antithesis of site fidelity, as if individuals tend to avoid the positions that they occupied in Episode 1. The stark contrast between this pattern and that of the previous example provides a clear illustration of how the shape of the landscape itself may have a substantive impact on expectations under a null model, patterns of inter-nest distances when individuals chose sites randomly.

In some circumstances, more than one method might be used to measure the distance between nests. For instance, the distance between the paired points in this example might be measured as an arc, rather than a chord, perhaps because the center region is for some reason avoided and individual movements are confined to the lake perimeter. If inter-nest distances in this example are computed as an arc, the histograms appear flat and distinct from the respective chord distances (Figure S2). Hence, as in this example, our *perception* of nest site fidelity, when based directly on patterns of inter-nest distances of repeat breeders, can be influenced by how distance is calculated. How distance is measured will generally be of less concern when an observed distribution is compared to a null model, where observed and simulated distances are calculated by the same method. In this example, for instance, arc and chord distances are monotonic functions of one another, so either measure provides the same information for comparison to a null model. But if, say, the nest site fidelity of a fish in a highly convoluted riparian system were of interest, then careful consideration may be required to determine how distance is best measured.

*Patchy landscape*

In the previous examples, nest habitat was assumed to be spatially continuous on a linear shoreline or around a lake perimeter. In many situations, suitable nest habitat may instead be found in discrete patches, separated by areas that are unsuitable for nests. For instance, individuals may breed in island colonies that are, in comparison to distances between nests within a colony, separated by very large distances, as occurs in terns and other sea birds [12].

Here, we portray such a scenario by the removal of the center third of the linear landscape described in Figure S1, so that suitable nest habitat is now located in two discrete patches that are separated by 120 arbitrary distance units. The null distributions of inter-nest distances in this situation are expected, based on 1,000 simulations, to be bimodal, whether suitable nest habitat is spatially homogeneous or concentrated in one of the two patches (Figure S3). How such a pattern is interpreted depends, however, on the spatial scale over which site fidelity is measured, as others have emphasized [2, 6, 7, 13, 14; see also 15]. The distribution of distances between the nests of individuals that bred in the same patch in both reproduction episodes—that is, all inter-nest distances less than 120 arbitrary units—is right skewed and, like the pattern observed for a homogeneous distribution of nest habitat on a single stretch of shoreline, creates the illusion of a choice process that involves some degree of site faithfulness. But the apparent fidelity to patches and overall site fidelity, as illustrated, is nearly equal to its expected outcome of 0.5.

The influence of scale on our perception of site fidelity is accentuated when nest habitat is concentrated in one of the two patches, where, in this illustration, six of the 13 repeat breeders that originated from the lower density [0, 120] patch in Episode 1 returned to the same patch in Episode 2, an apparent patch fidelity of 0.46 (Figure S3). Twenty-eight of the 37 repeat breeders that originated from the high density [240, 360] patch returned to the same patch in Episode 2, for an apparent patch fidelity of 0.76. The overall apparent patch fidelity in this illustration is 0.68. The expected apparent fidelity to the low-density and high-density patches, and overall, based on 1,000 simulations, are respectively 0.24, 0.75 and 0.62. Hence, the expected pattern of apparent site fidelity depends, as this example clearly shows, on an interaction between the distribution of nest habitat and the scale over which fidelity is measured.

**Behavioral Constraints**

The previous examples illustrated the sensitivity inter-nest distances of repeat breeders to the landscape and the distribution of suitable nest habitat under a null model. Behavior also influences how nests are distributed. Here, we modify selected previous examples to demonstrate some potential impacts of three behavioral constraints—territoriality, individual mobility, and habitat preferences—on expectations of inter-nest distances of repeat breeders under a null model.

*Territoriality*

First, consider how territoriality might influence the inter-nest distances of repeat breeders, where we again suppose that individuals nest on a linear landscape of 360 arbitrary units. For simplicity, imagine that all individuals build a nest at the center of an exclusive linear territory, where territory size—length—is invariant amongst breeders. The result is a distribution of nests within each reproduction episode that is relatively evenly spaced (Figure S4). The impact of territoriality on inter-nest distances, however, appears to be minimal when the histograms generated under this scenario are compared with those in Figure S1.

*Individual mobility*

Next, consider how restrictions on mobility can alter expectations of inter-nest distances of repeat breeders when, as in Figure S3, suitable nest habitat is located in two linear, discrete patches separated by 120 arbitrary distance units. Here, the movement of individuals is assumed to be limited, such that the inter-nest distance of a repeat breeder between reproduction episodes is constrained to no more than 240 distance units. In comparison to when movement is unrestricted, as depicted in Figure S3, the distribution is truncated and the right skewed portion of the distribution associated with individuals that remained in a patch is amplified (Figure S5). Indeed, the patch and overall fidelity when mobility is limited and suitable nest habitat is homogeneously distributed have an expectation, based on 1,000 simulations, of 0.69, an increase of 0.19 over conditions in which movement is unrestricted. When habitat is clumped the apparent faithfulness of individuals to the higher density patch is expected to be 0.83 and the expected return of repeat breeders to the lower density patch is just 0.39, with an overall expected patch fidelity of 0.72. The impact of limited mobility is an *apparent* increase in nest site faithfulness.

*Habitat preferences*

Lastly, consider a situation in which nests are built on a linear shoreline of 360 arbitrary distance units, where suitable nest habitat is divided into two types, one preferred over the other, with 50 nests of each type in Episode 1 and 2. In a null model, a nest preference is reflected in the order in which nests are assigned, preferred nests before nests that are less preferred. Hence, the impact of preferences on the distance between nests of repeat breeders will be most pronounced when they have an access advantage—that is, breed earlier than new breeders—to sites that are preferred. Here, we suppose that repeat breeders have an access advantage in both reproduction episodes, where the probability that a repeat breeder is assigned to a nest is five times that of a new breeder, as might occur when repeat breeders are, say, larger than new breeders (Appendix 2). The effect of this advantage is to bias the assignment of repeat breeders to nests that are preferred.

The relevant references against which to compare results for this scenario are the histograms in Figure S1. There is no impact of nest preferences on the distribution of inter-nest distances of repeat breeders when preferred and less preferred sites are distributed homogeneously on the landscape in both reproduction episodes (Figure S6). When preferred nests are spatially clumped, however, priority access to nests by repeat breeders produces a notable, strong right skew in the distribution of inter-nest distances due to their consistent use of space across reproduction episodes. Indeed, the potentially deceitful patterns that can be generated by a combination of habitat preferences, patchiness of preferred sites and priority access to sites by repeat breeders caution strongly against inferences about site faithfulness based solely on point patterns, without reference to a null model.

**Further Cautions**

The examples we developed uncover potential pitfalls associated with conjectures about site fidelity based solely on the distribution of distances between the nests of repeat breeders. Indeed, when distributions of inter-nest distances are not compared to a null model, hypotheses about the occurance of site fidelity may be falsely *accepted* or *rejected*. In some analyses, site fidelity might be inferred from a comparison of inter-nest distances between groups of individuals, where lack of reference to a null model may seem inconsequential. For instance, a win-stay, lose-shift strategy might be investigated, with the expectation that the distances between nests of individuals that breed successfully will be shorter—higher site fidelity—than those of individuals that are unsuccessful [25]. Likewise, foragers, like seabirds, may exhibit high site fidelity to areas in which abundant food is found [26, 27, 28; but see 9]. Alternatively, the overall variation in site fidelity within and among breeders or foragers might be contrasted, where higher between-individual than within-individual variation in site use is predicted when individuals are site faithful [29].

The examples we developed show that these approaches too are susceptible to inference errors when site-use patterns are not compared against a null model. For instance, if nest habitat is patchy and concentrated, say, in a core area where nests tend to be successful, then a null model in which individuals are assigned randomly to sites within and between patches can produce a pattern of site use that appears to reflect a win-stay, lose-shift strategy. The null model we devised for two patches, with nest habitat consistently, spatially clumped in one of the patches illustrates this very pattern: the expected patch fidelity of individuals with nests in the less dense patch in their first reproduction episode, where nest success may be poor, was 0.24, while the patch fidelity of individuals to the higher density, potentially higher quality patch was 0.75 (Figure S3). If suitable nest habitat is patchy, and individual mobility is limited, inferences based on within and between individual variability in site use may likewise benefit from null model safeguards (Figure S5).

**Application: More Sophisticated Null Models for *M. dolomieu***

For some systems, like ours, it is advisable to consider constraints on behavior that are not inherently imposed by the *recontre*-based procedure that we used to construct null models in the main paper. For instance, female *M. dolomieu* appear to preferentially spawn with males that build nests on coarse substrates, and egg survival in coarse substrate nests is often higher than in other types of nests [16, 17]. Hence, areas of the littoral zone composed of coarse substrates are likely preferred as locations for nests by male *M. dolomieu*. Recently used nests might also be preferred because of the potential consistency of good nest locations across years [18, 19]. The cost to excavate recently used nests may also be lower than construction costs for new nests. Indeed, a preference for suitable, recently used nests could, in principle, contribute to shorter distances between the nests of repeat breeders when nests are spatially clumped. Physical attributes, like body size, may also constrain when in a season an individual is able to breed, as in *M. dolomieu*, and because repeat breeders are generally older and larger than new breeders, they may often have an access advantage to nests over new breeders [see 16, 20, 21, 22, 23].

*Null models*

Nests in our dataset were categorized dichotomously as *coarse* or *other* and as *new* or *recently* *used*. Nests were classified as coarse if the substrate that covered the largest area of the bottom of the nest was composed of rock material 1 cm or larger in diameter [16]. Nests were defined as recently used in year *t* + 1 if a permanent marker of a nest used in year *t* was located within 1 m of the nest perimeter. Nests categorized as coarse or recently used were presumed to be preferred for the reasons we already mentioned.

To construct preference-based null models, we assigned preferred sites before other sites and constrained access to nests—the probability that a male was chosen and assigned to a nest—such that repeat breeders were never at a disadvantage to new breeders. This access asymmetry was imposed because in Pallette Lake repeat breeders tend to be larger than new breeders and, as observed in other systems, larger males tend to spawn earlier in a season than smaller males [22, 23]. In particular, we evaluated each preference-based null model under four access constraints, where repeat breeders and new breeders had equal access to sites or repeat breeders were two, five or 10 times as likely to gain access to a nest than new breeders (Appendix 2).

In each year, a few parental males were not captured, as indicated in Table 1 of the main paper. For the basic null model, their status as a new or repeat breeder had no impact on the nests to which known-status individuals were assigned and, hence, had no impact on simulated distances between the nests of repeat breeders. But the status of males that were not captured could impact nest assignments under null models that involve preferences and priority access to nests by repeat breeders. For consistency, uncaptured parental males were designated as repeat breeders in proportion to the ratio of repeat breeders amongst captured males in each year for all models, the base model included, and were assigned to a nest in accordance with any imposed preference or access rules. The straight-line distance between the nest assigned to each known repeat breeder in year *t* + 1 and the nest the individual actually occupied in year *t* was then computed and, as in the main paper, for each pair of years, 1,500 null inter-nest distance distributions were generated for comparison with the distribution that was observed. For each simulated distribution, we also recorded the number of repeat breeders assigned to the nest they previously occupied, as in the main paper, and the proportions of coarse substrate or recently used nests occupied by repeat breeders.

*Analyses*

The match between null model derived inter-nest distances for repeat breeders and observed distributions was evaluated by the same two approaches used in the main paper. In addition, we used the results from null models to investigate our assumptions about site preferences. Because repeat breeders should be distributed in proportion to the abundance of nests of different types when no preferences are exhibited, the proportion of nests occupied by repeat breeders should be identical for all nest types (Appendix 3). If, on the other hand, repeat breeders exhibit a preference for nests classified as *recently used* or *coarse*, as we supposed in our preference-based null models, and they have priority access to these nests, then the proportion of these nests occupied by repeat breeders in a given year should be high in comparison to their respective alternative types, *new* and *other*. Paired *t* tests were used to compare occupancy proportions for nests of different types, with the null hypothesis that the difference between yearly, paired proportions between any two nest types, averaged over all years is zero [24; Appendix 3].

*Results*

The majority ($\bar{X}\pm1 SE=0.75\pm0.01$) of nests in each season were constructed on coarse substrate and about half ($\bar{X}\pm1 SE=0.54\pm0.03$) of all nests had been used the previous year (Table S1). The proportion of coarse substrate nests occupied by repeat breeders in years 2002-2009 ranged between 0.35 and 0.70 ($\bar{X}\pm1 SE=0.57\pm0.04$). The *other* substrate nests were occupied by repeat breeders somewhat less frequently, in parital support of our assumption that coarse substrate nests are preferred ($\bar{X}\pm1 SE=0.48\pm0.07$; paired $t_{7}=2.02, P=0.0831$). However, the proportion of coarse substrate, recently used nests occupied by repeat breeders ($\bar{X}\pm1 SE=0.67\pm0.03$) was no higher than that of other substrate, recently used nests ($\bar{X}\pm1 SE=0.62\pm0.05$; paired $t_{7}=1.03, P=0.3393$) and no higher for coarse substrate, new nests ($\bar{X}\pm1 SE=0.42\pm0.05$) than for other substrate, new nests ($\bar{X}\pm1 SE=0.38\pm0.08$; paired $t_{7}=0.92, P=0.3881$).

Nest site occupancy by repeat breeders provided much stronger support for our assumption that recently used nests are preferred. The proportion of recently used nests occupied by repeat breeders ranged between 0.5 and 0.76 ($\bar{X}\pm1 SE=0.66\pm0.03$; Table S1). The proportion of new nests occupied by repeat breeders was significantly lower ($\bar{X}\pm1 SE=0.41\pm0.05$; paired $t_{7}=6.62, P=0.0002$). In addition, the proportion of recently used, coarse substrate nests occupied by repeat breeders ($\bar{X}\pm1 SE=0.67\pm0.03$) was far higher in comparison to new, coarse substrate nests ($\bar{X}\pm1 SE=0.42\pm0.05$; paired $t_{7}=7.11, P=0.0002$) and higher for recently used, other substrate nests ($\bar{X}\pm1 SE=0.62\pm0.05$) than for new, other substrate nests ($\bar{X}\pm1 SE=0.38\pm0.08$; paired $t_{7}=4.47, P=0.0029$).

The base null model replicated observed proportions of coarse substrate nests occupied by repeat breeders in five of the eight years even though no substrate preference was imposed, which suggests that the presumptive preference for coarse substrate nests is not especially powerful (Table S2). The proportion of recently used nests occupied by repeat breeders was matched by results of the base null model in just two years. Notably, the simulated proportions for the other six years were much smaller than those we observed, which provides further evidence that suitable sites that had been used in the previous year are preferred over suitable sites that were not recently used.

The preference-based null models better matched both the proportion of coarse substrate and recently used nests occupied by repeat breeders. In particular, the introduction of a preference for recently used nests and differential access to nests by repeat breeders into the base null model reproduced the observed proportion of recently used nests occupied by repeat breeders in every year and in seven of the eight years simultaneously replicated the proportion of coarse substrate nests occupied by repeat breeders (Table S3). The preference-based null models nonetheless performed no better than the basic null model with regard to the replication of distances between the successive nests of repeat breeders (Table S4, S5). Each of the 48,000 two-sample Kolmogorov-Smirnov tests resulted in a rejection of the null hypothesis that the simulated and observed distributions are identical at a significance level of $\alpha$ = 0.001. The lack of fit between the base null model, developed in the main paper, or any of these more sophisticated null models and the observed inter-nest distances of repeat breeders provides persuasive evidence that at least some males in the study population exhibited site fidelity.

**REFERENCES**

1. Pledger, S. and L. Bullen. 1998. Tests for Mate and Nest Fidelity in Birds with Application to Little Blue Penguins (*Eudyptula minor*). Biometrics 54, 61-65.

2. Wiegand, T. and K. A. Moloney. 2004. Rings, circles, and null-models for point pattern analysis in ecology. Oikos 104, 209-229.

3. Gotelli, N.J. and G. R. Graves, G.R. 1996. Null Models in Ecology. Smithsonian Institution Press, Washington, D. C.

4. Richardson, T. O., L. Giuggioli, N. R. Franks and A. B. Sendova-Franks. 2017. Measuring site fidelity and spatial segregation within animal societies. Methods in Ecology and Evolution 8, 965–975.

5. Cranmer, K., J. Brehmera and G. Louppec. 2020. The frontier of simulation-based inference. Procedings of the National Academy of Sciences 117, 30055–30062.

6. Schaefer, J. A., C. M. Bergman and S. N. Luttich. 2000. Site fidelity of female caribou at multiple spatial scales. *Landscape Ecology* 15, 731–739.

7. Campbell, S. P., J. W. Witham and M. L. Hunter, Jr. 2010. Stochasticity as an alternative to deterministic explanations for patterns of habitat use by birds. Ecological Monographs 80, 287-302.

8. Arthur, B., M. Hindell, M. Bester, P. Trathan, I. Jonsen, I. Staniland, W. C. Oosthuizen, M. Wege and M. A. Lea. 2015. Return customers: foraging site fidelity and the effect of environmental variability in wide-ranging Antarctic fur seals. PLoS ONE 10:e0120888.

9. Wakefield, E. D., I. R. Cleasby, S. Bearhop, T. W. Bodey, R. D. Davies, P. I. Miller, J. Newton, S. C. Votier and A. C. Hamer. 2015. Long-term individual foraging site fidelity—why some gannets don’t change their spots. Ecology 96, 3058–3074.

10. Borrmann R. M., R. A. Phillips, A. T. A. Clay and S. Garthe. 2019. High foraging site fidelity and spatial segregation among individual great black-backed gulls. Journal of Avian Biology https://doi.org/10.1111/jav.02156

11. R Core Team. 2021. R: A language and environment for statistical computing. R foundation for Statistical Computing, Vienna, Austria. URL https://www.R-project.org

12. Berthold, P. 2001. Bird Migration: A General Survey. Second Edition. Oxford University Press, New York.

13. Smogor, R. A., P. L. Angermeier and C. K. Gaylord. 1995. Distribution and Abundance of American Eels in Virginia Streams: Tests of Null Models across Spatial Scales. Transactions of the American Fisheries Society 124, 789-803.

14. Heap, S. M., D. Stuart-Fox and P. G. Byrne. 2015. Reduction in site fidelity with smaller spatial scale may suggest scale-dependent information use. Behavioral Ecology 26, 543–549.

15. Levin, S. A. 1992. The problem of pattern and scale in ecology. Ecology 73, 1943-1967.

16. Wiegmann, D. D., J. R. Baylis, and M. H. Hoff. 1992. Sexual selection and fitness variation in a population of smallmouth bass, *Micropterus dolomieui* (Pisces: Centrarchidae). Evolution 46, 1740–1753.

17. Saunders, R., M. A. Bozek, C. J. Edwards, M. J. Jennings and S. P. Newman. 2002. Habitat features affecting smallmouth bass *Micropterus dolomieu* nesting success in four northern Wisconsin lakes. American Fisheries Society Symposium 31, 123–134.

18. Rejwan, C., B. J. Shuter, M. S. Ridgway and N. C. Collins. 1997. Spatial and temporal distributions of smallmouth bass (*Micropterus dolomieu*) nests in Lake Opeongo, Ontario. Canadian Journal of Aquatic Sciences 54, 2007-2013.

19. Bozek, M. A., P. H. Short, C. J. Edwards, M. J. Jennings and S. P. Newman. 2002. Habitat selection of nesting smallmouth bass *Micropterus dolomieu* in two north temperate lakes. American Fisheries Society Symposium 31, 135–148.

20. Ridgway, M. S., B. J. Shuter and E. E. Post. 1991. The relative influence of body size and territorial behaviour on nesting asynchrony in male smallmouth bass, *Micropterus dolomieui* (Pisces: Centrarchidae). Journal of Animal Ecology 60, 665-681.

21. Baylis, J. R., D. D. Wiegmann and M. H. Hoff. 1993. Alternating life histories of smallmouth bass. Transactions of the American Fisheries Society 122, 500-510.

22. LaRoche, R. A. S., K. L. Weinersmith, M. L. Davis, L. A. Angeloni, J. R. Baylis, S. P. Newman, S. P. Egan and D. D. Wiegmann. 2023. Size-associated energetic constraints on the seasonal onset of reproduction in a species with indeterminate growth. Oikos https://doi.org/10.1111/oik.09739.

23. Davis, M. L. 2021. Does Experience Influence Nest Characteristics or Timing of Reproduction by Male Smallmouth Bass (*Micropterus dolomieu*)? [Master's thesis, Bowling Green State University]. OhioLINK Electronic Theses and Dissertations Center. http://rave.ohiolink.edu/etdc/view?acc_num=bgsu1626977908215714

24. Glover, T. and K. Mitchell. 2016. An Introduction to Biostatistics. Third Edition. Long Grove, IL. Waveland Press.

25. Robert, A, V. H. Paiva, M. Bolton, F. Jiguet and J. Bried J. 2014 Nest fidelity is driven by multi-scale information in a long-lived seabird. Proceedings of the Royal Society B 281: 20141692. dx.doi.org/10.1098/rspb.2014.1692

26. Carroll, G., R. Harcourt, B. J. Pitcher, D. Slip and I. Jonsen. 2018. Recent prey capture experience and dynamic habitat quality mediate short-term foraging site fidelity in a seabird. Proceedings of the Royal Society B: Biological Sciences, 285, 20180788. doi.org/10.1098/rspb.2018.0788.

27. Beal, M., P. Byholm, U. Lötberg, T. J. Evans, K. Shiomi and S. Åkesson. 2021. Habitat selection and foraging site fidelity in Caspian Terns (*Hydroprogne caspia*) breeding in the Baltic Sea. Ornis Fennica 98, 128–141.

28. Bonnet-Lebrun, A-S, J. Collet, R. A. Phillips. 2021. A test of the win-stay—lose-shift foraging strategy and its adaptive value in albatrosses. Animal Behaviour 182, 145-151.

29. Hertel, A. G., P. T. Niemelä, N. J. Dingemanse, and T. Mueller*.* 2020*.* A guide for studying among-individual behavioral variation from movement data in the wild.  Movement Ecology 8, 30. doi.org/10.1186/s40462-020-00216-8

**FIGURES**

**Figure S1.** Relationship between the distribution of suitable nest habitat and inter-nest distances of repeat breeders when individuals choose nest locations randomly on a hypothetical, linear shoreline of length 360 arbitrary units in two reproduction episodes. Dot plots show 100 randomly chosen points—nest locations—in the interval [0, 360] when (*top*) suitable nest habitat is uniformly distributed or (*bottom*) clumped, with 50 of the 100 sites in the interval [135, 225]. The upper and lower rows represent the sites occupied in Episode 1 and Episode 2, respectively and solid dots show the nest locations of the 50 repeat breeders in Episode 2. Lines project back to their respective nest locations in Episode 1. Histograms summarize the expected frequencies ($\bar{X}\pm2 SE)$ of inter-nest distances, based on 1,000 simulations, between paired nest locations of repeat breeders in Episodes 1 and 2.

**Figure S2.** Relationship between the distribution of suitable nest habitat and inter-nest distances of repeat breeders in two reproduction episodes when individuals choose nest locations randomly on a circular landscape with a circumference of 360 arbitrary units. Points are identical to those in Figure S1, where the linear landscape is now wrapped into a circle with a circumference of 360 arbitrary units. For clarity, nest locations are shown only for repeat breeders. The solid dots are locations of repeat breeders in Episode 2. Lines—chords—trace back to their nest locations, the open dots, in Episode 1. Histograms summarize the expected frequencies ($\bar{X}\pm2 SE)$ of inter-nest distances, based on 1,000 simulations, between the paired nest sites of repeat breeders in Episodes 1 and 2, where distance is measured as the chord or the shortest arc around the circle perimeter that connects their nest positions.


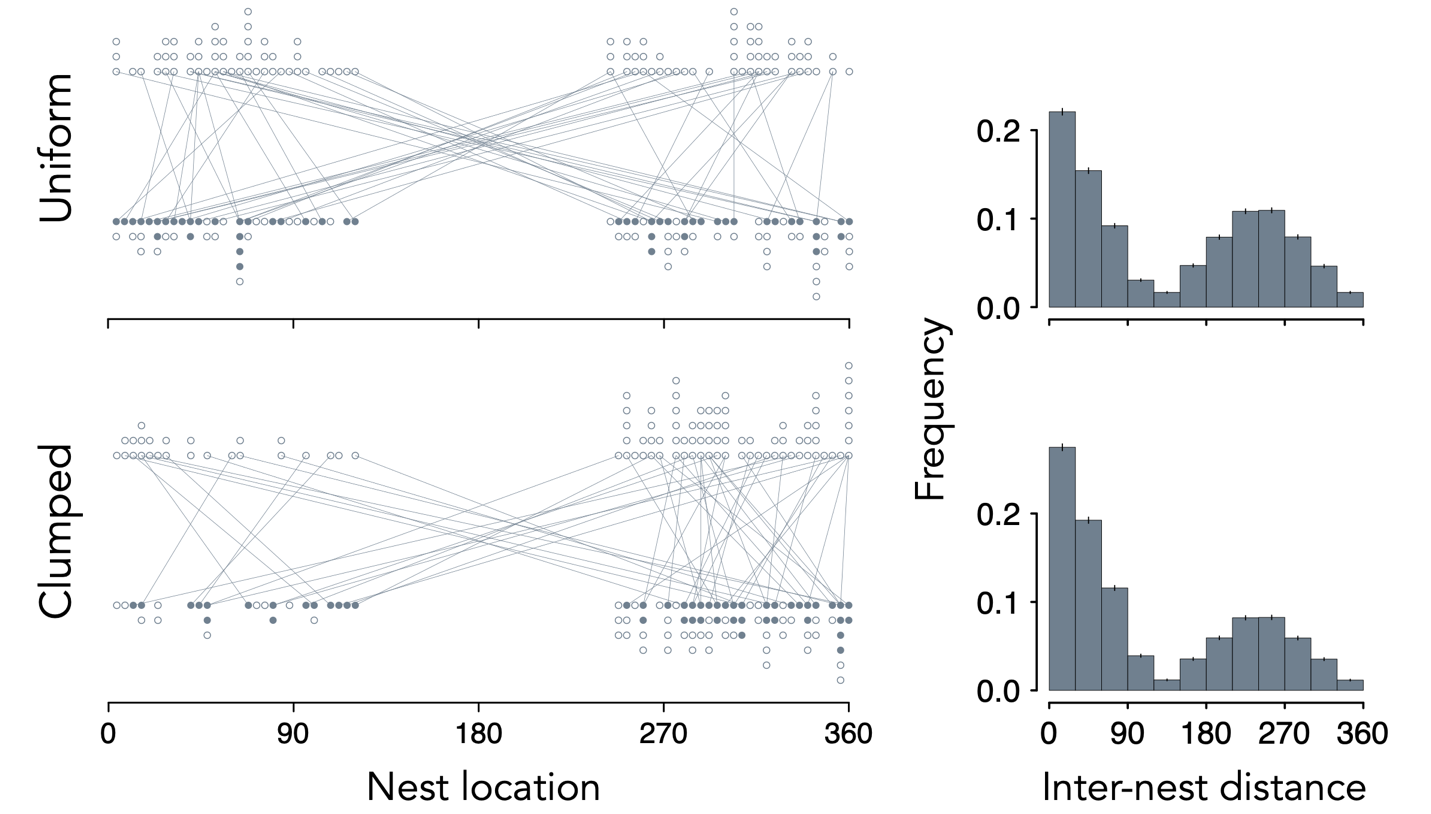


**Figure S3.** Relationship between the distribution of suitable nest habitat and inter-nest distances of repeat breeders between two reproduction episodes when individuals choose nest locations randomly from two patches 120 arbitrary units in length on a linear stretch of shoreline 360 units in length, where patches are separated by 120 units. Dot plots show 100 randomly chosen points—nest locations—in the intervals [0, 120] and [240, 360] when (*top*) suitable nest habitat is uniformly distributed in the two intervals or (*bottom*) concentrated in one of the patches, with 75 of the 100 sites in the interval [240, 360]. Dots in the upper and lower rows represent nest sites in Episode 1 and Episode 2 and solid dots indicate the nest locations of the 50 repeat breeders in Episode 2. Lines project back to their nest locations in Episode 1. Histograms summarize the expected frequencies ($\bar{X}\pm2 SE)$ of distances, based on 1,000 simulations, between nest locations of individual repeat breeders in Episodes 1 and 2.

**Figure S4.** Inter-nest distances of territorial repeat breeders, where individuals choose nest locations randomly in two reproduction episodes on a shoreline of 360 arbitrary units. Dot plots show 100 random points—nest locations—on the interval [0, 360] when suitable nest habitat is uniformly distributed or clumped, with 50 of the 100 sites in the interval [135, 225] in Episode 1 and Episode 2. No nests are closer than 1 arbitrary unit due to territoriality. The solid dots are the nest locations of the 50 repeat breeders in Episode 2. Lines trace back to their nest locations in Episode 1. Histograms summarize the expected frequencies ($\bar{X}\pm2 SE)$ distances, based on 1,000 simulations, between the paired nest locations of individual repeat breeders in Episodes 1 and 2.

**Figure S5.** Inter-nest distances of repeat breeders between two reproduction episodes when individuals choose nest locations randomly from two patches 120 arbitrary units in length on a linear stretch of shoreline 360 units in length, where patches are separated by 120 units and individual movements are limited, such that the distance between nests can be no farther than 240 arbitrary units. Dot plots show 100 randomly chosen points—nest locations—in the intervals [0, 120] and [240, 360] when suitable nest habitat is uniformly distributed or clumped, with 75 of the 100 sites in the interval [240, 360]. Dots in the upper and lower rows represent nest sites in Episode 1 and Episode 2 and solid dots indicate the nest locations of the 50 randomly chosen repeat breeders in Episode 2. Lines project back to their nest locations in Episode 1. Histograms summarize the expected frequencies ($\bar{X}\pm2 SE)$ of inter-nest distances, based on 1,000 simulations, between nest locations of individual repeat breeders in Episodes 1 and 2.

**Figure S6.** Relationship between the distribution of nest habitat on a hypothetical stretch of linear shoreline 360 arbitrary units in length and inter-nest distances of repeat breeders when individuals exhibit habitat preferences and repeat breeders have an access advantage to nests. Dot plots show 100 randomly chosen points—nest locations—when 50 preferred sites are uniformly distributed in the interval [0, 360] or clumped in the interval [135, 225]. Dots, the less preferred sites, and triangles, the preferred sites, in the upper and lower rows represent the locations of nests occupied in Episode 1 and Episode 2. Filled dots and triangles are the nest locations of the 50 randomly chosen repeat breeders in Episode 2. Lines project back to their nest locations in Episode 1. Histograms summarize the expected frequencies ($\bar{X}\pm2 SE)$ of distances, based on 1,000 simulations, between nest locations of individual repeat breeders in Episodes 1 and 2.

**TABLES**

**Table S1.** Number of nests by category (coarse substrate or other; recently used or new) in which females spawned and occupancy rates by repeat breeders, 2002-2009. Margin totals are in bold.

|  |  | Occupancy | | | | | | |
| --- | --- | --- | --- | --- | --- | --- | --- | --- |
|  |  | Total | | |  | Repeat breeders | | |
| Year |  | Recent | New |  |  | Recent | New |  |
| 2002 | Coarse | 92 | 90 | **182** |  | 47 | 18 | **65** |
|  | Other | 20 | 39 | **59** |  | 9 | 5 | **14** |
|  |  | **112** | **129** |  |  | **56** | **23** |  |
|  |  |  |  |  |  |  |  |  |
| 2003 | Coarse | 114 | 98 | **212** |  | 76 | 42 | **118** |
|  | Other | 31 | 25 | **56** |  | 20 | 4 | **24** |
|  |  | **145** | **123** |  |  | **96** | **44** |  |
|  |  |  |  |  |  |  |  |  |
| 2004 | Coarse | 145 | 109 | **254** |  | 86 | 36 | **122** |
|  | Other | 21 | 54 | **75** |  | 9 | 15 | **24** |
|  |  | **166** | **163** |  |  | **95** | **51** |  |
|  |  |  |  |  |  |  |  |  |
| 2005 | Coarse | 116 | 51 | **167** |  | 85 | 32 | **117** |
|  | Other | 31 | 44 | **75** |  | 17 | 25 | **44** |
|  |  | **147** | **95** |  |  | **102** | **57** |  |
|  |  |  |  |  |  |  |  |  |
| 2006 | Coarse | 99 | 54 | **153** |  | 69 | 21 | **90** |
|  | Other | 31 | 30 | **61** |  | 22 | 15 | **37** |
|  |  | **130** | **84** |  |  | **91** | **36** |  |
|  |  |  |  |  |  |  |  |  |
| 2007 | Coarse | 57 | 65 | **122** |  | 41 | 40 | **81** |
|  | Other | 17 | 31 | **48** |  | 17 | 20 | **37** |
|  |  | **74** | **96** |  |  | **58** | **60** |  |
|  |  |  |  |  |  |  |  |  |
| 2008 | Coarse | 82 | 75 | **157** |  | 63 | 29 | **92** |
|  | Other | 18 | 36 | **54** |  | 10 | 6 | **16** |
|  |  | **100** | **111** |  |  | **73** | **35** |  |
|  |  |  |  |  |  |  |  |  |
| 2009 | Coarse | 127 | 41 | **168** |  | 85 | 17 | **102** |
|  | Other | 24 | 23 | **47** |  | 18 | 13 | **31** |
|  |  | **151** | **64** |  |  | **103** | **30** |  |

**Table S2.** Proportions of coarse substrate nests (CSN) and recently used nests (RUN) occupied by repeat breeders and simulated proportions under the base null model. The observed (*O*) values of statistics not in an extreme tail of the distribution of simulated statistics are indicated in bold. Expected (*E*) values are based on 1,500 simulations under the basic null model. Ω is the frequency of the 1,500 simulated proportions that deviated more in absolute value from *E* than the difference between *E* and *O*.

|  | CSN | | |  | RUN | | |
| --- | --- | --- | --- | --- | --- | --- | --- |
| Year | *O* | *E* | Ω |  | *O* | *E* | Ω*** |
| 2002 | 0.36 | **0.33** | 0.1073 |  | 0.50 | 0.33 | < 0.0007 |
| 2003 | 0.56 | **0.53** | 0.0960 |  | 0.66 | 0.53 | < 0.0007 |
| 2004 | 0.48 | 0.44 | 0.0187 |  | 0.57 | 0.44 | < 0.0007 |
| 2005 | 0.70 | 0.61 | 0.0367 |  | 0.69 | **0.61** | 0.1480 |
| 2006 | 0.59 | **0.59** | 0.8820 |  | 0.70 | 0.59 | < 0.0007 |
| 2007 | 0.66 | **0.68** | 0.4487 |  | 0.76 | **0.68** | 0.0713 |
| 2008 | 0.59 | 0.51 | 0.0007 |  | 0.73 | 0.51 | < 0.0007 |
| 2009 | 0.61 | **0.62** | 0.6060 |  | 0.68 | 0.62 | 0.0047 |

* Ω < 0.0007 indicates that the observed proportion was not contained in the distribution of proportions generated in 1,500 simulations.

**Table S3.** Expected proportions (*E*) of coarse substrate nests (CSN) and recently used nests (RUN) occupied by repeat breeders when null models include a preference for either CSN or RUN. Expected proportions are means based on 1,500 simulations under four nest access constraints (Bias: 1, 2, 5, 10).* Bolded *E* are the means of simulated distributions in which the observed proportion was contained.**

|  |  | Preference | | | | | | | | | | |
| --- | --- | --- | --- | --- | --- | --- | --- | --- | --- | --- | --- | --- |
|  |  | CSN | | | | |  | RUN | | | | |
|  |  | CSN | |  | RUN | |  | CS | |  | RUN | |
| Year | Bias | *E* | Ω |  | *E* | Ω |  | *E* | Ω |  | *E* | Ω |
| 2002 | 1 | **0.34** | 0.4073 |  | 0.33 | < 0.0007 |  | **0.32** | 0.1233 |  | 0.34 | < 0.0007 |
|  | 2 | 0.40 | 0.0020 |  | 0.35 | < 0.0007 |  | **0.34** | 0.2553 |  | 0.45 | 0.1293 |
|  | 5 | 0.43 | < 0.0007 |  | 0.36 | < 0.0007 |  | **0.35** | 0.5747 |  | **0.58** | 0.0033 |
|  | 10 | 0.43 | < 0.0007 |  | 0.36 | < 0.0007 |  | **0.35** | 0.8207 |  | 0.66 | < 0.0007 |
|  |  |  |  |  |  |  |  |  |  |  |  |  |
| 2003 | 1 | 0.59 | 0.0020 |  | 0.53 | < 0.0007 |  | 0.53 | 0.0953 |  | 0.59 | 0.0033 |
|  | 2 | 0.64 | < 0.0007 |  | 0.53 | < 0.0007 |  | **0.53** | 0.0727 |  | **0.68** | 0.4047 |
|  | 5 | 0.67 | < 0.0007 |  | 0.53 | < 0.0007 |  | 0.53 | 0.0227 |  | 0.79 | < 0.0007 |
|  | 10 | 0.67 | < 0.0007 |  | 0.53 | < 0.0007 |  | 0.53 | 0.0107 |  | 0.85 | < 0.0007 |
|  |  |  |  |  |  |  |  |  |  |  |  |  |
| 2004 | 1 | 0.50 | 0.0447 |  | 0.47 | < 0.0007 |  | 0.45 | 0.0500 |  | 0.50 | 0.0133 |
|  | 2 | 0.55 | < 0.0007 |  | 0.49 | < 0.0007 |  | **0.47** | 0.3480 |  | **0.61** | 0.1373 |
|  | 5 | 0.57 | < 0.0007 |  | 0.50 | 0.0047 |  | **0.48** | 1.0000 |  | 0.73 | < 0.0007 |
|  | 10 | 0.57 | < 0.0007 |  | 0.50 | 0.0040 |  | **0.49** | 0.3213 |  | 0.80 | < 0.0007 |
|  |  |  |  |  |  |  |  |  |  |  |  |  |
| 2005 | 1 | 0.66 | 0.0440 |  | **0.66** | 0.1793 |  | 0.66 | 0.0460 |  | **0.66** | 0.1620 |
|  | 2 | **0.72** | 0.4027 |  | **0.68** | 0.5833 |  | **0.67** | 0.1860 |  | **0.73** | 0.2120 |
|  | 5 | 0.79 | < 0.0007 |  | **0.70** | 0.8753 |  | **0.69** | 0.6360 |  | 0.80 | < 0.0007 |
|  | 10 | 0.83 | < 0.0007 |  | **0.71** | 0.4047 |  | **0.70** | 1.0000 |  | 0.84 | < 0.0007 |
|  |  |  |  |  |  |  |  |  |  |  |  |  |
| 2006 | 1 | **0.60** | 0.2540 |  | 0.59 | 0.0007 |  | **0.59** | 0.8827 |  | 0.60 | 0.0007 |
|  | 2 | 0.67 | < 0.0007 |  | 0.61 | < 0.0007 |  | **0.60** | 0.5253 |  | **0.68** | 0.4780 |
|  | 5 | 0.74 | < 0.0007 |  | 0.62 | 0.0013 |  | **0.61** | 0.2913 |  | 0.77 | 0.0120 |
|  | 10 | 0.79 | < 0.0007 |  | 0.62 | 0.0007 |  | **0.61** | 0.1393 |  | 0.81 | < 0.0007 |
|  |  |  |  |  |  |  |  |  |  |  |  |  |
| 2007 | 1 | **0.69** | 0.2540 |  | **0.68** | 0.0940 |  | **0.68** | 0.4647 |  | **0.69** | 0.1293 |
|  | 2 | 0.75 | < 0.0007 |  | **0.70** | 0.1767 |  | **0.69** | 0.3587 |  | **0.77** | 0.7353 |
|  | 5 | 0.82 | < 0.0007 |  | **0.71** | 0.1847 |  | **0.69** | 0.2500 |  | 0.85 | 0.0053 |
|  | 10 | 0.86 | < 0.0007 |  | **0.72** | 0.2740 |  | **0.69** | 0.1727 |  | 0.89 | < 0.0007 |
|  |  |  |  |  |  |  |  |  |  |  |  |  |
| 2008 | 1 | 0.52 | 0.0020 |  | 0.52 | < 0.0007 |  | 0.51 | < 0.0007 |  | 0.52 | < 0.0007 |
|  | 2 | **0.59** | 0.7127 |  | 0.54 | < 0.0007 |  | 0.52 | 0.0027 |  | 0.63 | 0.0040 |
|  | 5 | 0.67 | < 0.0007 |  | 0.56 | < 0.0007 |  | 0.53 | 0.0027 |  | **0.76** | 0.3327 |
|  | 10 | 0.69 | < 0.0007 |  | 0.56 | < 0.0007 |  | 0.54 | 0.0107 |  | 0.83 | < 0.0007 |
|  |  |  |  |  |  |  |  |  |  |  |  |  |
| 2009 | 1 | 0.69 | < 0.0007 |  | 0.64 | 0.0333 |  | **0.63** | 0.1720 |  | **0.69** | 0.7627 |
|  | 2 | 0.73 | < 0.0007 |  | **0.65** | 0.0820 |  | 0.64 | 0.0527 |  | 0.74 | 0.0020 |
|  | 5 | 0.77 | < 0.0007 |  | **0.66** | 0.2353 |  | 0.65 | 0.0007 |  | 0.80 | < 0.0007 |
|  | 10 | 0.79 | < 0.0007 |  | **0.67** | 0.2593 |  | 0.66 | < 0.0007 |  | 0.84 | < 0.0007 |

*Bias: 1, 2, 5 and 10 indicate that repeat breeders were equally, twice, five or ten times as likely to be assigned to a nest as a new breeder.

** The observed proportions for each year are found in Table S2. Ω < 0.0007 indicates that the observed proportion was not contained in the distribution of proportions generated in 1,500 simulations.

**Table S4.** Expected inter-nest distance statistics based on 1,500 simulations in which a preference for coarse substrate nests was imposed. Bolded values of variances indicate that the observed variance of distances was contained in the distribution of the 1,500 simulated variances (Ω > 0.05).* *Bias* is the nest access advantage given to repeat breeders, where one indicates that repeat and new breeders had equal access to nests.** *Proportion* indicates the frequency of repeat breeders expected to occupy the same exact location in consecutive years.

|  |  | Statistics | | | | | |
| --- | --- | --- | --- | --- | --- | --- | --- |
| Episodes | Bias | Mean | Median | Variance*** | Skew | Kurtosis | Proportion |
| 2001-2002 | 1 | 585 | 630 | **89,260** | -0.27 | 1.92 | 0.0009 |
|  | 2 | 582 | 624 | **88,823** | -0.27 | 1.93 | 0.0009 |
|  | 5 | 579 | 622 | **88,774** | -0.27 | 1.92 | 0.0010 |
|  | 10 | 580 | 623 | **88,755** | -0.27 | 1.92 | 0.0010 |
|  |  |  |  |  |  |  |  |
| 2002-2003 | 1 | 582 | 622 | 91,375 | -0.23 | 1.87 | 0.0004 |
|  | 2 | 580 | 618 | 91,430 | -0.23 | 1.86 | 0.0003 |
|  | 5 | 578 | 616 | 91,372 | -0.22 | 1.86 | 0.0004 |
|  | 10 | 579 | 615 | 91,386 | -0.23 | 1.86 | 0.0004 |
|  |  |  |  |  |  |  |  |
| 2003-2004 | 1 | 570 | 607 | **86419** | -0.21 | 1.91 | 0.0003 |
|  | 2 | 567 | 601 | **85987** | -0.20 | 1.92 | 0.0003 |
|  | 5 | 565 | 598 | **86089** | -0.20 | 1.91 | 0.0004 |
|  | 10 | 564 | 597 | **86283** | -0.20 | 1.91 | 0.0004 |
|  |  |  |  |  |  |  |  |
| 2004-2005 | 1 | 582 | 630 | **86338** | -0.27 | 1.96 | 0.0003 |
|  | 2 | 583 | 632 | **85869** | -0.28 | 1.97 | 0.0003 |
|  | 5 | 582 | 629 | **85101** | -0.28 | 1.97 | 0.0003 |
|  | 10 | 581 | 628 | **85123** | -0.28 | 1.97 | 0.0003 |
|  |  |  |  |  |  |  |  |
| 2005-2006 | 1 | 590 | 640 | 86891 | -0.31 | 2.00 | 0.0009 |
|  | 2 | 588 | 639 | 85979 | -0.30 | 1.99 | 0.0009 |
|  | 5 | 585 | 633 | 85448 | -0.30 | 1.99 | 0.0010 |
|  | 10 | 583 | 630 | 85079 | -0.30 | 1.98 | 0.0010 |
|  |  |  |  |  |  |  |  |
| 2006-2007 | 0 | 584 | 630 | 85172 | -0.28 | 2.00 | 0.0005 |
|  | 2 | 579 | 624 | 84358 | -0.27 | 1.99 | 0.0005 |
|  | 5 | 577 | 622 | 83616 | -0.27 | 1.98 | 0.0003 |
|  | 10 | 576 | 618 | 83190 | -0.26 | 1.98 | 0.0004 |
|  |  |  |  |  |  |  |  |
| 2007-2008 | 1 | 561 | 600 | 84127 | -0.22 | 1.93 | 0.0014 |
|  | 2 | 558 | 593 | 83784 | -0.21 | 1.91 | 0.0017 |
|  | 5 | 556 | 589 | 83526 | -0.21 | 1.90 | 0.0015 |
|  | 10 | 555 | 587 | 83238 | -0.21 | 1.90 | 0.0017 |
|  |  |  |  |  |  |  |  |

**Table S4** (continued)

|  |  | Statistics | | | | | | |
| --- | --- | --- | --- | --- | --- | --- | --- | --- |
| Episodes | Bias | Mean | Median | Variance*** | Skew | Kurtosis | | Proportion |
| 2008-2009 | 1 | 560 | 600 | 83134 | -0.26 | 1.90 | | 0.0017 |
|  | 2 | 558 | 598 | 83332 | -0.25 | 1.89 | | 0.0016 |
|  | 5 | 555 | 595 | 83386 | -0.25 | 1.88 | | 0.0017 |
|  | 10 | 554 | 592 | 83460 | -0.24 |  | 1.87 | 0.0017 |

* The observed values of statistics are found in Table 2 of the primary paper.

** Please see Appendix 2 for a precise definition of the nest access advantage to repeat breeders.

*** The observed values of all distance statistics, except the variance, were not contained in any of the 1,500 simulated distributions generated for each access bias (Ω < 0.0007). The row-wise Ω for bolded variances are 0.4800, 0.4967, 0.5067, 0.5307 (2001-2002); 0.4733, 0.5233, 0.4847, 0.4647 (2003-2004); and 0.1973, 0.2080, 0.2533, 0.2720 (2004-2005).

**Table S5.** Expected inter-nest distance statistics based on 1,500 simulations in which a preference for recently used nests was imposed. Bolded values of variances indicate that the observed variance was contained in the distribution of the 1,500 simulated variances (Ω > 0.05).* *Bias* is the nest access advantage given to repeat breeders, where one indicates that repeat and new breeders had equal access to nests.** *Proportion* indicates the frequency of repeat breeders expected to occupy the same exact location in consecutive years.

|  |  | Statistics | | | | | |
| --- | --- | --- | --- | --- | --- | --- | --- |
| Episodes | Bias | Mean | Median | Variance*** | Skew | Kurtosis | Proportion |
| 2001-2002 | 1 | 586 | 631 | **89418** | -0.27 | 1.92 | 0.0008 |
|  | 2 | 583 | 627 | **89850** | -0.27 | 1.92 | 0.0010 |
|  | 5 | 582 | 628 | **89189** | -0.28 | 1.92 | 0.0014 |
|  | 10 | 580 | 627 | **89043** | -0.28 | 1.93 | 0.0016 |
|  |  |  |  |  |  |  |  |
| 2002-2003 | 1 | 582 | 621 | 91710 | -0.23 | 1.87 | 0.0004 |
|  | 2 | 580 | 619 | 91627 | -0.22 | 1.86 | 0.0005 |
|  | 5 | 579 | 617 | 92213 | -0.22 | 1.85 | 0.0006 |
|  | 10 | 578 | 615 | 92248 | -0.22 | 1.85 | 0.0006 |
|  |  |  |  |  |  |  |  |
| 2003-2004 | 1 | 575 | 614 | **87524** | -0.22 | 1.92 | 0.0004 |
|  | 2 | 573 | 611 | **88260** | -0.21 | 1.90 | 0.0004 |
|  | 5 | 571 | 608 | **89266** | -0.20 | 1.88 | 0.0005 |
|  | 10 | 570 | 606 | **89718** | -0.20 | 1.87 | 0.0006 |
|  |  |  |  |  |  |  |  |
| 2004-2005 | 1 | 583 | 632 | **86291** | -0.28 | 1.97 | 0.0003 |
|  | 2 | 582 | 631 | **86449** | -0.28 | 1.96 | 0.0003 |
|  | 5 | 581 | 628 | **86389** | -0.27 | 1.95 | 0.0004 |
|  | 10 | 580 | 628 | **86129** | -0.27 | 1.95 | 0.0003 |
|  |  |  |  |  |  |  |  |
| 2005-2006 | 1 | 591 | 642 | 87018 | -0.31 | 1.99 | 0.0009 |
|  | 2 | 592 | 642 | 87181 | -0.31 | 1.99 | 0.0010 |
|  | 5 | 593 | 643 | 87387 | -0.31 | 1.99 | 0.0011 |
|  | 10 | 592 | 641 | 87566 | -0.30 | 1.98 | 0.0013 |
|  |  |  |  |  |  |  |  |
| 2006-2007 | 0 | 583 | 629 | 85158 | -0.28 | 1.99 | 0.0005 |
|  | 2 | 584 | 631 | 85256 | -0.28 | 2.00 | 0.0006 |
|  | 5 | 584 | 630 | 86222 | -0.28 | 1.98 | 0.0007 |
|  | 10 | 584 | 631 | 86175 | -0.28 | 1.99 | 0.0007 |
|  |  |  |  |  |  |  |  |
| 2007-2008 | 1 | 561 | 598 | 84444 | -0.22 | 1.93 | 0.0016 |
|  | 2 | 559 | 593 | 84255 | -0.21 | 1.92 | 0.0019 |
|  | 5 | 555 | 589 | 84509 | -0.19 | 1.91 | 0.0022 |
|  | 10 | 552 | 583 | 84873 | -0.18 | 1.90 | 0.0025 |
|  |  |  |  |  |  |  |  |
|  |  |  |  |  |  |  |  |

**Table S5** (continued)

|  |  | Statistics | | | | | | |
| --- | --- | --- | --- | --- | --- | --- | --- | --- |
| Episodes | Bias | Mean | Median | Variance*** | Skew | Kurtosis | | Proportion |
|  |  |  |  |  |  |  | |  |
| 2008-2009 | 1 | 561 | 602 | 83371 | -0.25 | 1.91 | | 0.0018 |
|  | 2 | 561 | 602 | 83400 | -0.26 | 1.91 | | 0.0020 |
|  | 5 | 559 | 600 | 83868 | -0.25 | 1.90 | | 0.0021 |
|  | 10 | 560 | 601 | 83697 | -0.25 |  | 1.91 | 0.0024 |

* The observed values of statistics are found in Table 2 of the primary paper.

** Please see Appendix 2 for a precise definition of the nest access advantage to repeat breeders.

*** The observed values of all distance statistics, except the variance, were not contained in any of the 1,500 simulated distributions generated for each access bias (Ω < 0.0007). The row-wise Ω for bolded variances are 0.4860, 0.4633, 0.4947, 0.5033 (2001-2002); 0.3820, 0.3373, 0.2253, 0.2153 (2003-2004); and 0.2080, 0.1807, 0.1793, 0.2023 (2004-2005).

**Appendix 1**

Here, we provide the essential R code used to generate null distributions of inter-nest distances in our examples. The initial step was to set the seed so that numbers could be replicated.

set.seed(1) # use this seed to exactly reproduce the results

**Ecological Constraints**

*Linear landscape*

For the situation depicted in Figure S1 and Figure 1 of the main paper, where suitable habitat is uniformly distributed on a line in two reproduction episodes, we drew two sets of 100 points between 0 and 360 from a uniform distribution and assigned the points randomly to 100 individuals in Episode 1 (x1) and Episode 2 (x2) where 50 arbitrarily chosen individuals from Episode 1 were designated as repeat breeders and randomly assigned to a location in Episode 2. The code we used is

df_Episode_1 <- data.frame(x1 = runif(100, 0, 360), Repeat_breeder_1 = sample(c(rep(1, 50), rep(0, 50)), 100, replace = FALSE))

df_Episode_2 <- data.frame(x2 = runif(100, 0, 360, Repeat_breeder_2 = sample(c(rep(1, 50), rep(0, 50)), 100, replace = FALSE))

The data frames were then subset to include only repeat breeders (Repeat_breeder_1 == 1, Repeat_breeder_2 == 1) and their randomly assigned positions (x1, x2) were extracted. The code that corresponds to df_Episode_1 is

df_1 <- subset(df_Episode_1, Repeat_breeder_1 == 1)

x1 <- df_1$x1

The absolute difference between the paired points (x1, x2) was then computed to determine inter-nest distances:

inter_nest_distance <- abs(x2 - x1) # compute distances

To simulate a situation in which nests are clumped in the interval [135, 225] we needed to sample 50 positions uniformly from [0, 135] and [225, 360]. The code here is written so that any two intervals [*a*, *b*] and [*c*, *d*] (*a* < *b* < *c* < *d*) might be explored, where for Figure 1 of the main paper we used *a* = 0, *b* = 135, *c* = 225 and *d* = 360 to generate the 50 points:

# episode 1 [0, 135] and [225, 360]

y <- runif(1, a, b - a + d - c) # pick a number [0, 270]

if(y < (b - a)) { # if the number is less than 135 assign that position

x1 <- a + y

} else {

x1 <- c + y - (b - a) # if y >= 135 assign position 225 + (y – 135)

}

while (length(x1) < 50) { # add 49 more points to x1

y <- runif(1, a, b - a + d - c)

if(y < (b - 0)) {

x <- a + y

} else {

x <- c + y - (b - 0)

}

x1 <- append(x1, x)

}

Next, we appended 50 randomly chosen positions in the clumped interval [135, 225] to x1

# episode 1 [135, 225]

while (length(x1) < 100) {

x <- runif(1, 135, 225)

x1 <- append(x1, x)

}

and created a data frame as before:

df_Episode_1 <- data.frame(x1, Repeat_breeder_1 = sample(c(rep(1, 50),

rep(0, 50)), 100, replace = FALSE))

The data frame was then subset as already described and the x1 positions for repeat breeders were extracted. (This procedure imposed an order on the points in x1—points in the interval [135, 225] occur after points in the other two intervals—but because Repeat_breeder_1 was randomly ordered there was no need to shuffle x1 before the data frame was created.) The same procedures were used to generate df_Episode_2 and positions (x2) of repeat breeders in Episode 2, and the distances between the nests of designated repeat breeders were computed as earlier. This code was looped 1,000 times to generate the histograms in Figure S1 and Figure 1 of the main paper.

*Circular landscape*

The boundaries [0, 360] were purposely chosen so that points could also be envisioned as locations in units of degrees on the perimeter of a circle with a circumference of 360 arbitrary units, equal in length to our imaginary stretch of beach or river. To wrap the points used in Figure 1 of the main paper onto a circle with a circumference of 360 units, as we did in Figure S2, we converted x1 and x2, now viewed as degrees, into *x* and *y* coordinates on the perimeter of the circle:

radius <- 360/(2*pi) # radius of circle with circumference 360 units

df_Episode_1$x_1 <- radius*cos(x1*pi/180) # find x coordinate of x1

df_Episode_1$y_1 <- radius*sin(x1*pi/180) # find y coordinate of x1

df_Episode_2$x_2 <- radius*cos(x2*pi/180) # find x coordinate of x2

df_Episode_2$y_2 <- radius*sin(x2*pi/180) # find y coordinate of x2

The straight-line, chord distances between the paired points were then computed as

chord <- sqrt((df_Episode_2$x2 - df_Episode_1$x1)^2 + (df_Episode_2$y2 - df_Episode_1$y1)^2)

To compute the minimum arc distance between two points, we first used

theta <- acos(1 - chord^2/(2*rad^2))

to determine the angle between points (in radians) and then computed the arc as

arc <- rad*theta

The arc here is the shortest distance that connects two points around the circle and, hence, cannot exceed 180 arbitrary units.

*Patchy landscape*

The code used to simulate the patchy landscape in Figure S3, where nests are located in the intervals [0, 120] and [240, 360], is virtually identical to the code used to generate Figure 1 of the main paper. To generate a uniform distribution of nests for x1 (or x2) over the two patches, we borrowed the code used earlier to clump nests in the interval [135, 225], where habitat boundaries were set to *a* = 0, *b* = 120, *c* = 240 and *d* = 360 to sample 100 numbers. To generate positions for the situation with 75 nests clumped in the interval [240, 360] we used

x1 = sample(c(runif(25, 0, 120), runif(75, 240, 360), 100, replace = FALSE)

**Behavioral Constraints**

Here, we provide the essential code for the three behavioral constraints we considered. Histograms were generated by 1,000 iterations of the code in each of the cases.

*Territoriality*

In this scenario, depicted in Figure S4, we again supposed that nests are constructed on a linear stretch of shoreline of length 360 arbitrary units, where in both reproduction episodes at least 1 arbitrary unit separated all nests.

To simulate a situation in which nests are uniformly distributed in Episode 1, we first picked a random location on the line:

x1 <- runif(1, 0, 360) # sample random number from [0, 360]

Next, we appended 99 numbers in the interval [0, 360] to x1 with the restriction that each added number was at least 1 arbitrary unit from all other points in x1:

while (length(x1) < 100) {

x <- runif(1, 0, 360) # sample random number from a uniform distribution [0, 360]

d = abs(x1 - x) # absolute distance between chosen number and points in x1

if (min(d) >= 1) { # add the chosen number if >= 1 from all other nests

x1 <- append(x1, x)

}

}

Then, as before, the data frame df_Episode_1 was generated and x1 was extracted for repeat breeders. The same procedures were used to create the data frame for Episode 2 and, as earlier, the distances between paired points of repeat breeders were computed.

To simulate a situation in which individuals are territorial and nests are clumped in a core area, we simply added the restrictions on d, as just shown, to the code we used earlier to clump nests, without territoriality, in the interval [135, 225].

*Limited mobility*

In this scenario, depicted in Figure S5, we adapted our straight-line, two-patch model, where individual movement was limited such that the maximum distance between the nests of an individual repeat breeder was constrained to not farther than 240 arbitrary units. For the situation in which nests are uniformly distributed on the intervals [0, 120] and [240, 360], the data frame for Episode 1 (df_Episode_1) was constructed exactly as it was for the patch model described earlier. In Episode 2, the movement restriction constrained where the 50 nests of the repeat breeders could be located, while the locations of nests of new breeders were unrestricted. The code used to assign the 50 new breeders in Episode 2 is identical to that used for Episode 1. To assign repeat breeders to nests in Episode 2 (x2) and restrict movement so that the two nests of each individual are not more than 240 arbitrary units apart, we iterate through df_Episode_1, renamed here to df to save space:

for (i in 1:nrow(df)) {

if (df[i, "x1"] <= 120) { # fist location is in patch 1 [0, 120]

# choose location in patch 1 or between 240 and first location + 240

y <- runif(1, 0, 120 - 0 + (df[i, "x1"] + 240) - 240)

if (y < (120 - 0)) { # if y is in patch 1 [0, 120]

df[i, "x2"] = 0 + y # assigned location is y

} else {

df[i, "x2"] = 240 + y - (120 - 0) # y > 120 and assign to y + 240

}

}

else { # fist location is in patch 2 [240, 360]

# choose location in patch 2 or between first location – 240 and 120

y <- runif(1, 0, 120 - (df[i, "x1"] - 240) + 360 - 240)

if (y < (120 - (df[i, "x1"] - 240))) { # y is in patch 1

df[i, "x2"] = (df[i, "x1"] - 240) + y # assigned location in patch 1

} else { # y is in patch 2

df[i, "x2"] = 240 + y - (120 - (df[i, "x1"] - 240)) # assigned point

}

}

}

The inter-nest distances are then computed as the absolute difference between the data frame columns x1 and x2.

To simulate a situation in which nest habitat is clumped, with 25 nests in the interval [0, 120] and 75 nests in the interval [240, 360], we needed to modify this code to restrict how many individuals were assigned to the two patches. The assignment of individuals to nests in Episode 1 was simple:

df_Episode_1 <- data.frame(x1 = sample(c(runif(25, 0, 120), runif(75, 240,

360)), 100, replace = FALSE), Repeat_breeder_1 = sample(c(rep(0, 50),

rep(1, 50)), 100, replace = FALSE))

For convenience, we used the column Repeat_breeder_1 to control the order in which new and repeat breeders were assigned to nest locations in Episode 2 and simply added a column (x2) to the data frame to indicate positions. (The x1 and x2 for rows of df_Episode_1 assigned to new breeders in Episode 1 (Repeat_breeder_1 == 0) are not paired and x2 simply denotes the location of new breeders in Episode 2.) The first step was to set the number of occupants of the two patches to zero in Episode 2:

patch_1 = 0

patch_2 = 0

Then, we modified the code for the situation in which nests were uniformly distributed in the two patches so that patch occupancy could be controlled, where, to save space, the data frame is again renamed to df:

for (i in 1:nrow(df)) { # iterate through rows of df

if (df[i, "Repeat_breeder_1"] == 0) { # row corresponds to new breeder

if (patch_1 < 25 & patch_2 < 75) { # neither patch is full

y <- runif(1, 0, 120 - 0 + 360 - 240) # pick number between 0 and 240

if (y < (120 - 0)) { # y is less than 120

df[i, "x2"] = 0 + y # chosen location is y

} else {

df[i, "x2"] = 240 + y - (120 - 0) # y > 120 and point is y + 120

}

}

else if (patch_1 == 25) { # patch 1 is full

df[i, "x2"] = runif(1, 240, 360) # choose random location in patch 2

}

else {

df[i, "x2"] = runif(1, 0, 120) # patch 2 full, choose point in patch 1

}

}

if (df[i, "Repeat_breeder_1"] == 1) { # row of a repeat breeder

if (patch_1 < 25 & patch_2 < 75) { # neither patch is full

if (df[i, "x1"] <= 120) { # fist location is in patch 1

# choose location in patch 1 or between 240 and first location + 240

y <- runif(1, 0, 120 - 0 + (df[i, "x1"] + 240) - 240)

if (y < (120 - 0)) { # y is in patch 1

df[i, "x2"] = 0 + y # assigned location is y

} else {

df[i, "x2"] = 240 + y - (120 - 0) # assigned point in patch 2 }

}

else { # fist location is in patch 2

# choose location in patch 2 or between first location – 240 and 120

y <- runif(1, 0, 120 - (df[i, "x1"] - 240) + 360 - 240)

if (y < (120 - (df[i, "x1"] - 240))) { # y is in patch 1

df[i, "x2"] = (df[i, "x1"] - 240) + y # assigned location is y

} else {

df[i, "x2"] = 240 + y - (120 - (df[i, "x1"] - 240)) # point chosen

}

}

}

else if (patch_1 == 25) { # patch 1 is full

if (df[i, "x1"] <= 120) { # fist location is in patch 1

# choose random location in patch 2 within 240 limit

df[i, "x2"] = runif(1, 240, df[i, "x1"] + 240)

} else { # nest in Episode 1 in patch 2

df[i, "x2"] = runif(1, 240, 360) # choose random location in patch 2

}

}

else { # patch 2 is full

if (df[i, "x1"] <= 120) { # fist location is in patch 1

df[i, "x2"] = runif(1, 0, 120) # choose random location in patch 1

} else { # first nest in patch 2

# choose random location in patch 1 within limit of 240

df[i, "x2"] = runif(1, df[i, "x1"] - 240, 120)

}

}

}

if (df[i, "x2"] <= 120) { # update patch 1 occupancy

patch_1 = patch_1 + 1

}

else { # update patch 2 occupancy

patch_2 = patch_2 + 1

}

}

The data frame was subset to include only rows for repeat breeders (Repeat_breeder_1 == 1) and the absolute distance between x1 and x2 was then computed.

*Habitat preferences*

In this scenario, depicted in Figure S6, we again used our shoreline model, where now two types of nests in equal abundance were envisioned, one type preferred over the other. Here, individuals were assigned to nests in a first come, first served procedure, repeat breeders had an access advantage over new breeders and individuals were not assigned to nests of the less preferred type until all nests of the preferred type were occupied.

The first step was to determine the order in which new breeders and repeat breeders gained access to nests in Episode 1, where the probability *p* that an individual repeat breeder was chosen was always five times that of a new breeder, *q* (Appendix 2). The code we used to control these probabilities and the order of assignments of new (Repeat_breeder_1 == 0) and repeat breeders (Repeat_breeder_1 == 1) to 50 preferred nests, which were uniformly distributed on the landscape, was:

Number_repeat_breeders_1 = 50 # set number of future repeat breeders, R

Number_new_breeders_1 = 50 # set number of new breeders, N

Higher_quality_nest_assignments_1 <- vector() # empty vector

# assign N and R to preferred nests, with p = 5q

for (i in 1:50) { # fill 50 preferred nests

draw = runif(1, 0, 1) # draw a random number [0, 1]

p = 1/(Number_repeat_breeders_1 + Number_new_breeders_1/2) # set probably

if (draw <= p*Number_repeat_breeders_1) { # R gets preferred nest

Higher_quality_nest_assignments_1 <-

c(Higher_quality_nest_assignments_1, 1)

Number_repeat_breeders_1 = Number_repeat_breeders_1 - 1 # reduce R

}

else { # otherwise, assign N to a high quality nest

Higher_quality_nest_assignments_1 <-

c(Higher_quality_nest_assignments_1, 0)

Number_new_breeders_1 = Number_new_breeders_1 - 1 # reduce N

}

}

Next, we assign the remainder of the individuals to less preferred nests and generate a uniform distribution of nest locations. (Note that the order of assignment does not matter.) The code is:

# fill 50 less preferred nests with remainder of R and N

Lower_quality_nest_assignments_1 <- sample(c(rep(1,

Number_repeat_breeders_1), rep(0, Number_new_breeders_1)), replace =

FALSE)

x1 <- runif(100, 0, 360) # uniform distribution of nest locations

Now, we stack the assignment vectors to create Repeat_breeder_1 and create a data frame df_episode_1 to indicate which males were assigned to preferred nests. Finally, we subset the data frame to isolate repeat breeders, df_1:

df_episode_1 <- data.frame(x1, Repeat_breeder_1, quality_1 = c(rep(1, 50),

rep(0, 50))) # create data frame with first 50 nests preferred

df_1 <- subset(df_episode_1, Repeat_breeder_1 == 1) # subset for R

The same code was used to generate a complimentary data frame df_episode_2 for Episode 2. Because repeat breeders that were assigned to the preferred (less preferred) nests all appear at the top (end) of the x1 and x2 columns of df_1 and df_2 it was necessary to shuffle df_1 or df_2 before the rows of points were paired. To do this we randomly sampled the rows of df_2 and reordered the data frame:

Index_repeat_2 <- sample(nrow(df_2), 50, replace = FALSE)

df_2 <- df_2[Index_repeat_2, ] # re-order data frame

The absolute difference between x1 and x2 was then computed.

For a situation in which 50 preferred nests are clumped in the interval [135, 225] in Episode 1, with 50 less-preferred nests distributed uniformly in the intervals [0, 135] and [225, 360], the code is identical, expect for how x1 and x2 were generated. For x1 and x2 we first sampled the 50 preferred nests. The code for x1 is:

# episode 1 [135, 225]

x1 <- runif(50, 135, 225)

The code used to draw 50 points from the two intervals [0, 135] and [225, 360] is identical to the code we used for the clumped scenario in Figure 1 of the main paper.

**Appendix 2**

Here, we show how the order in which repeat and new breeders are assigned to nests can be controlled so as to maintain a constant, fixed access advantage for repeat breeders. Let $N$ equal the total number of breeders, with $N_{1}$ repeat breeders and $N_{2}$ new breeders, and suppose that the individuals in the two groups have respective assignment probabilities $p_{1}$ and $p_{2}$. If all breeders have equal access to nest sites, then at any instant $p_{1}=p_{2}=p=1/N$ and $N_{1}p_{1}+N_{2}p_{2}=N_{1}p+N_{2}p=p\left( N_{1}+N_{2} \right)=pN=1$.

These relations can be used to adjust $p_{1}$ and $p_{2}$ to introduce an access advantage to nests by repeat breeders. If repeat breeders are, for example, twice as likely to gain access to a nest than new breeders, then $p_{1}=2p_{2}$ and $N_{1}p_{1}+N_{2}{p_{1}}/2=1$. Hence, the probability that any particular repeat breeder will be assigned to a nest is $p_{1}=1/\left( N_{1}+{N_{2}}/2 \right)$. To control the assignment of individuals to nests, we draw a random number $a$ from a uniform [0, 1] distribution and if $a\leq N_{1}p_{1}$ a repeat breeder is randomly chosen and randomly assigned to a nest, subject to any constraints. Otherwise, a new breeder is assigned. The number of repeat breeders or new breeders is then decreased by one and $p_{1}=1/\left( N_{1}+{N_{2}}/2 \right)$ is recalculated with the revised $N_{1}$ or $N_{2}$ to maintain the access advantage $p_{1}=2p_{2}$. In our analyses of preference-based null models we imposed $p_{1}=p_{2}$, $p_{1}=2p_{2}$, $p_{1}=5p_{2}$ and $p_{1}=10p_{2}$ and refer to the access afforded to repeat breeders as an access bias that equals the $p_{2}$ coefficient (*i*.*e*., 1, 2, 5 or 10).

**Appendix 3**

If nests of different types are chosen randomly, then nest occupancy by repeat breeders should be in proportion to the abundance of the different nest types. For instance, if there are *R* repeat breeders and *n* nest types, with abundances $t_{1}, t_{2}, t_{3}, \ldots, t_{n}$, where $T=t_{1}+t_{2}+\ldots+t_{n}$, then the proportion of repeat breeders expected to occupy nests of type *i* is $p_{i}={t_{i}}/T$. If nests of type *i* are preferred and repeat breeders have an access advantage to preferred nests, then the observed proportion of repeat breeders on type *i* nests should be larger than ${t_{i}}/T$.

When there are more than two nest types it is useful, for comparative purposes, to focus on the *proportion of nests* of a particular type that are occupied by repeat breeders, say $q_{i}$, rather than on the proportion of repeat breeders that occupy nests of type *i*. The expected proportion of nests of any type *i* that will be occupied by repeat breeders if sites are chosen randomly is ${q_{i}=\left[ \left( {t_{i}}/T \right)R \right]}/{t_{i}}=R/T$. Because this proportion is a constant and independent of nest type, the expected difference between proportions of nests of any two types *i* and *j* occupied by repeat breeders when there are no nest type preferences is zero. This result was the foundation of our analyses of nest preferences with paired *t* tests. In particular, the yearly, paired $q_{i}-q_{j}$ when averaged across all years of the study should yield a paired *t* = 0 if nests of type *i* and *j* are equally preferred.
