# Supplementary figures and images for: Null models confirm nest site fidelity by male smallmouth bass, Micropterus dolomieu
Source: BMC Zool. 2024 Jun 27;9:13. doi: 10.1186/s40850-024-00205-z (PMC11210175; doi:10.1186/s40850-024-00205-z)

Uniform

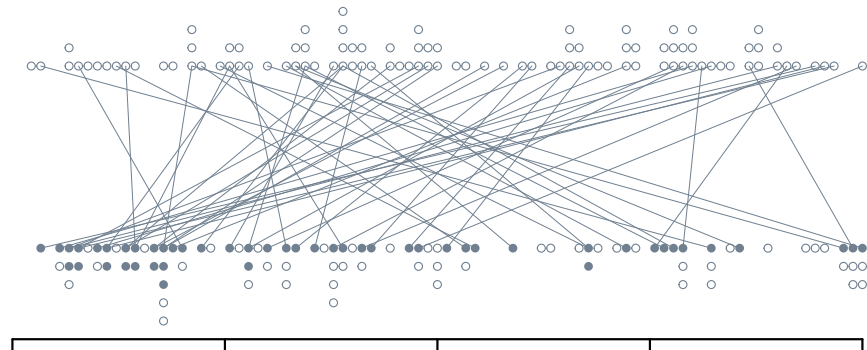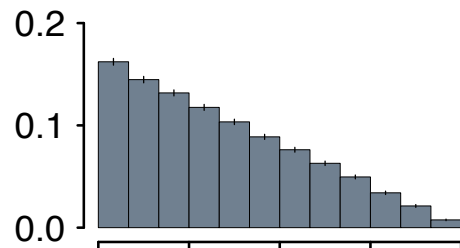

Clumped

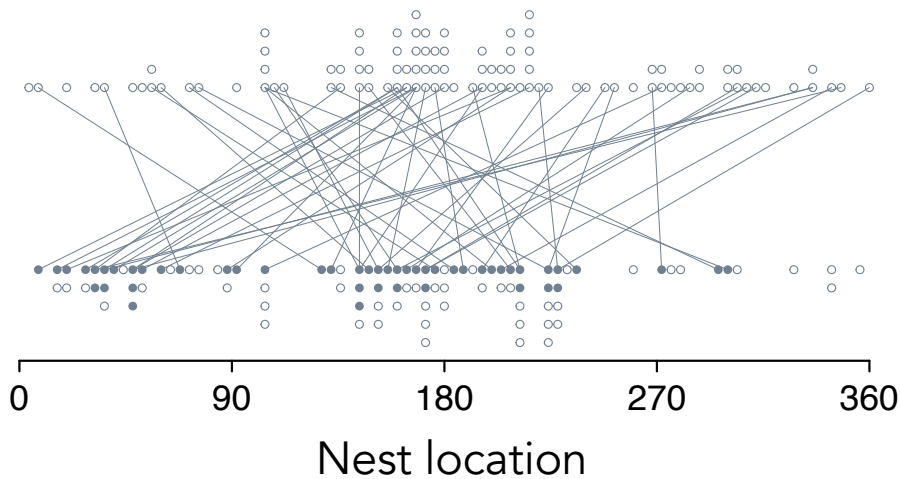

Frequency

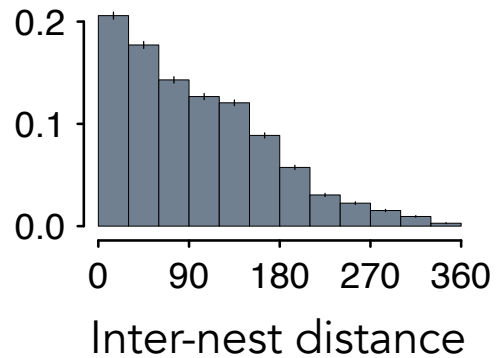

Supplement: Supplementary file 2 — Supplementary Material 2. [file 40850_2024_205_MOESM2_ESM.pdf]

Uniform

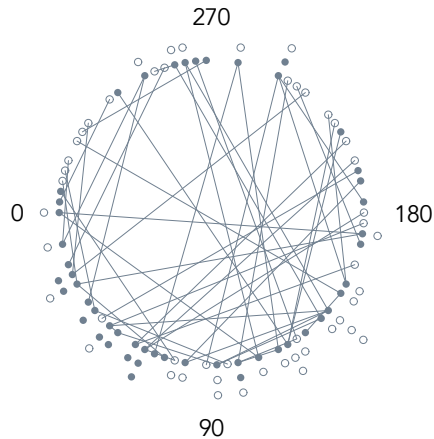

Frequency

Chord

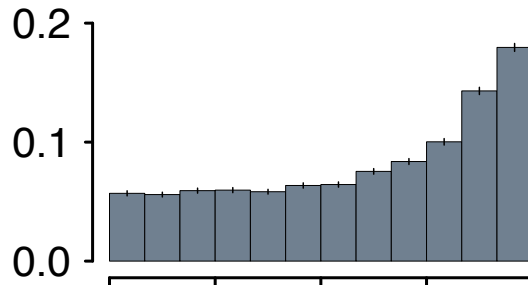

Arc

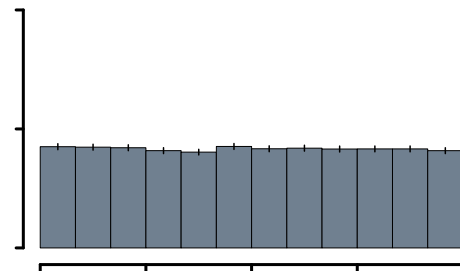

Clumped

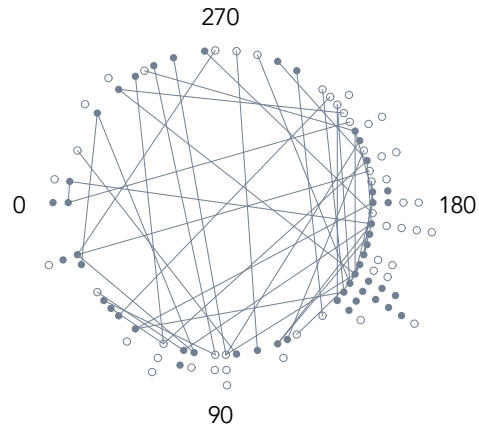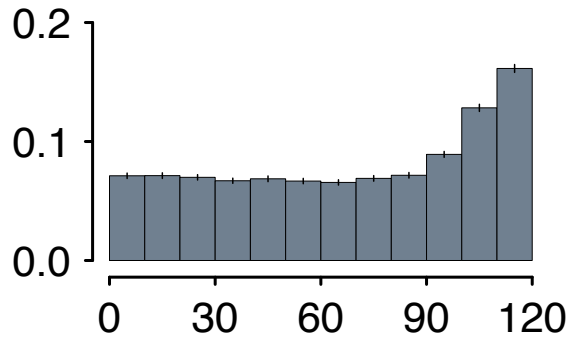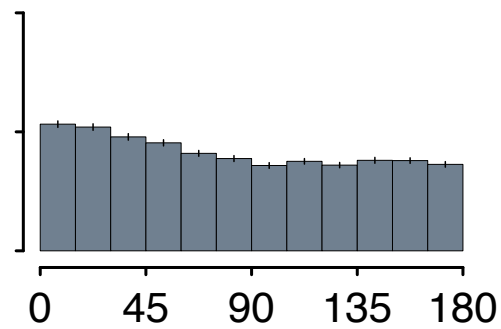

Inter-nest distance

Supplement: Supplementary file 3 — Supplementary Material 3. [file 40850_2024_205_MOESM3_ESM.pdf]

Uniform

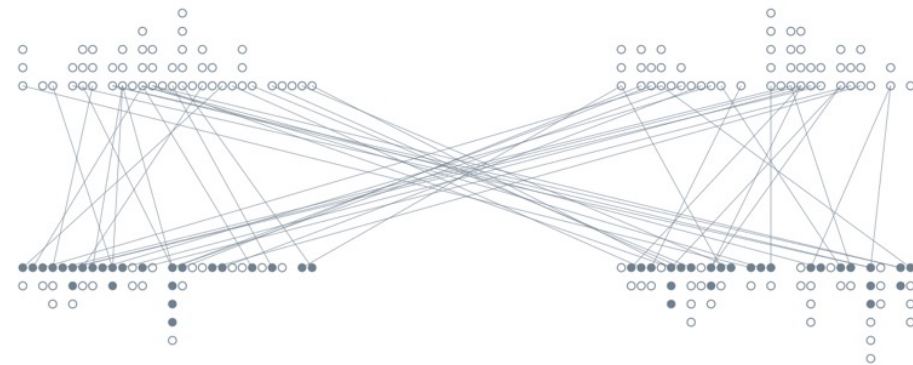

0.2

0.1

0.0

Frequency

0.2

0.1

0.0

0 90 180 270 360

Inter-nest distance

Clumped

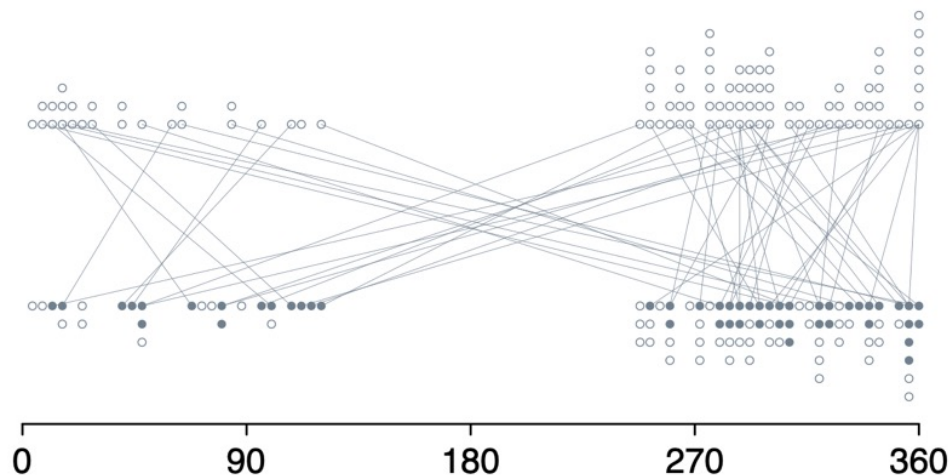

Nest location

Supplement: Supplementary file 4 — Supplementary Material 4. [file 40850_2024_205_MOESM4_ESM.pdf]

Uniform

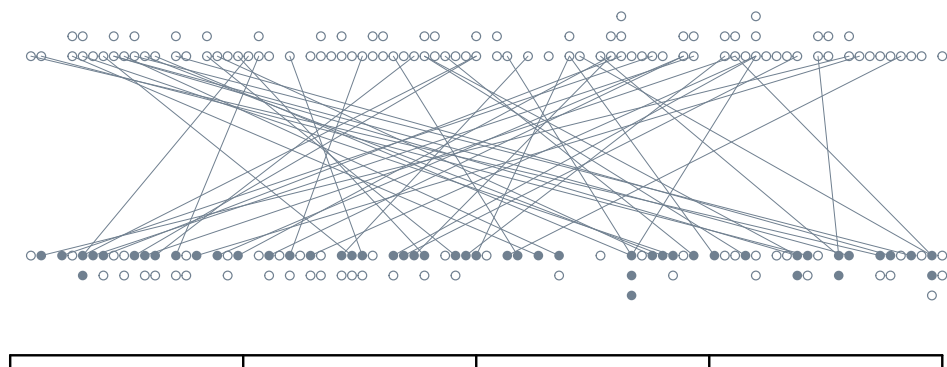

Frequency

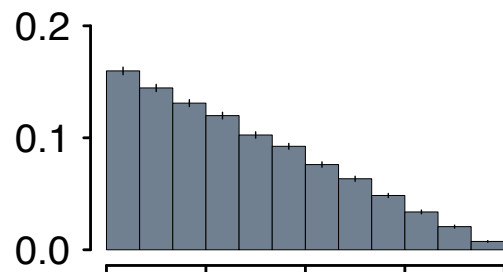

Clumped

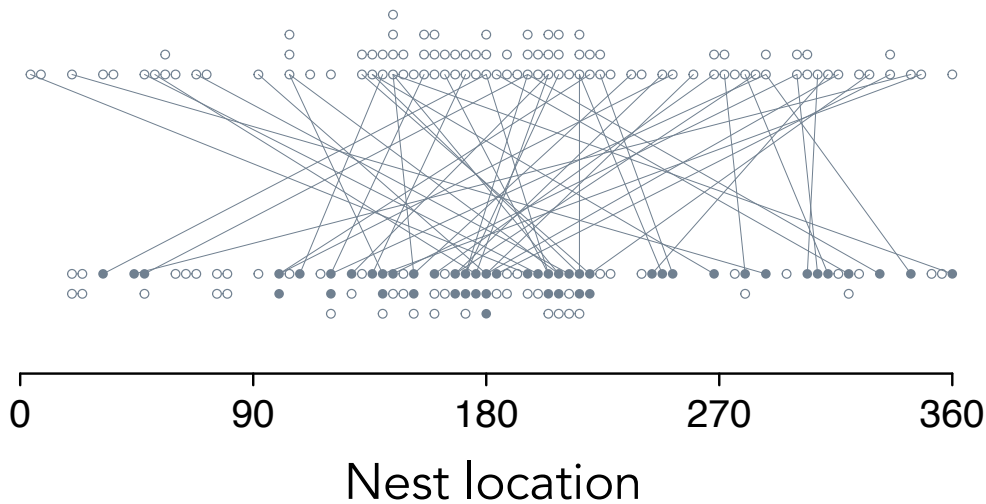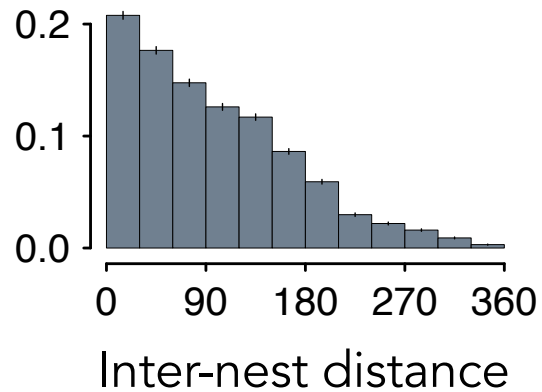

Supplement: Supplementary file 5 — Supplementary Material 5. [file 40850_2024_205_MOESM5_ESM.pdf]

Uniform

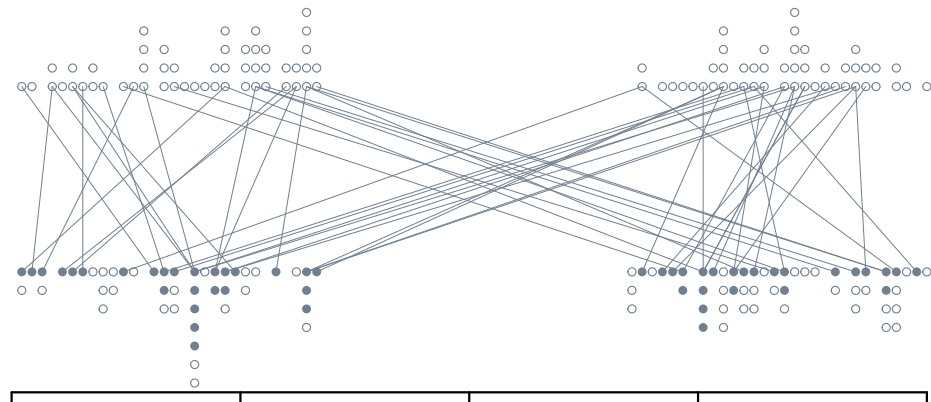

Frequency

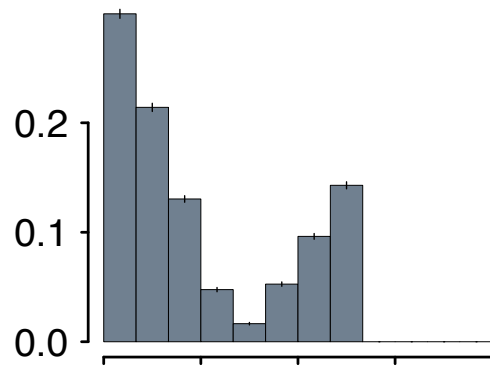

Clumped

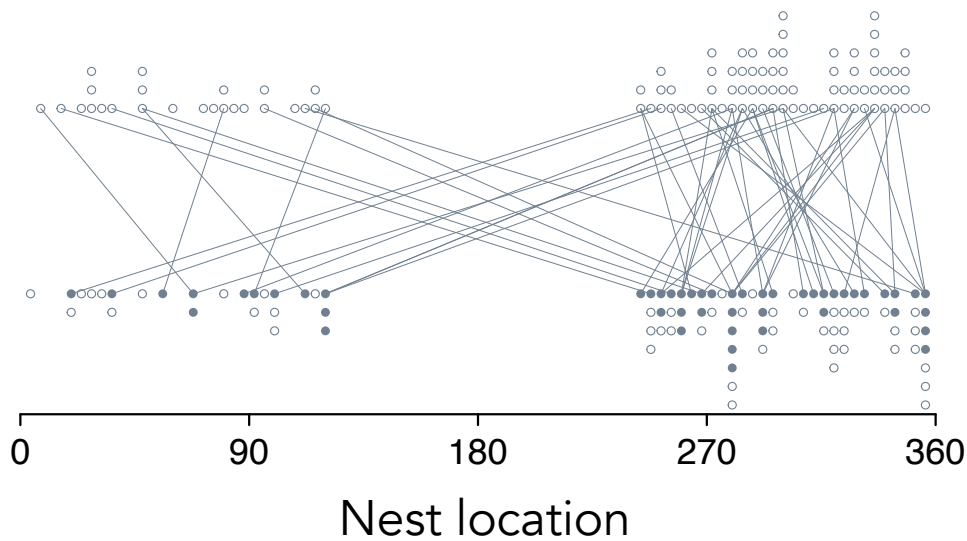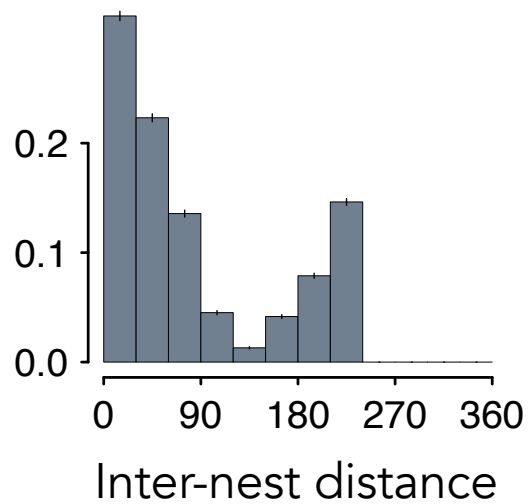

Supplement: Supplementary file 6 — Supplementary Material 6. [file 40850_2024_205_MOESM6_ESM.pdf]

Uniform

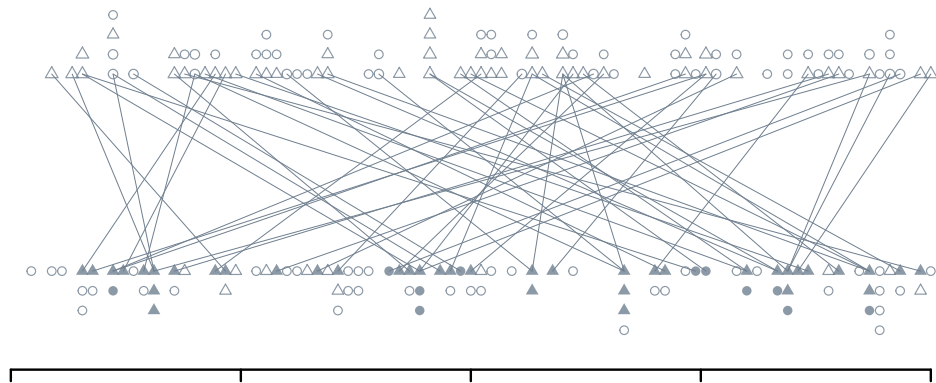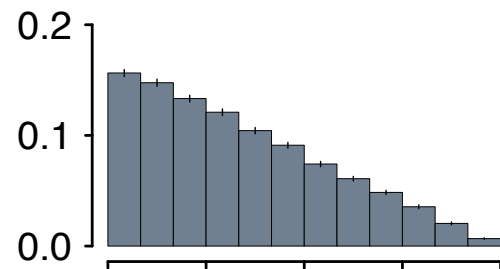

Clumped

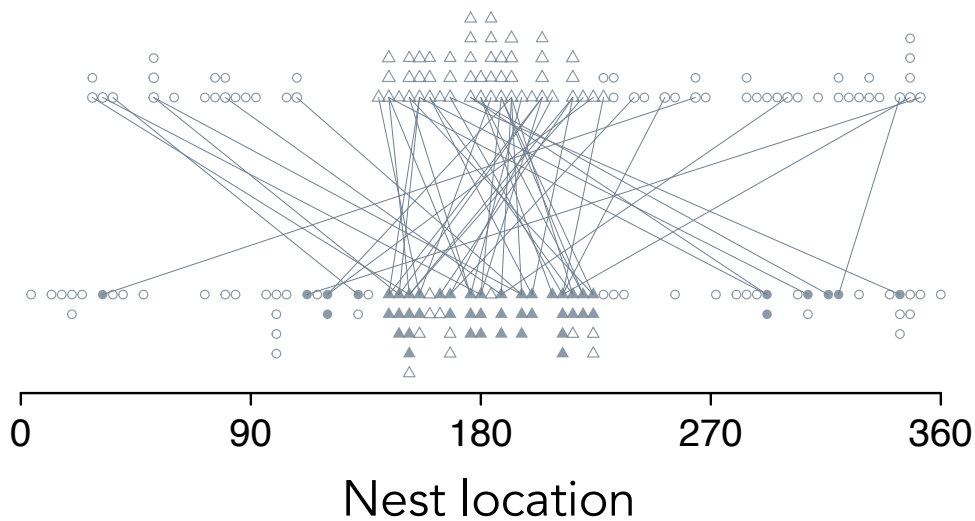

Frequency

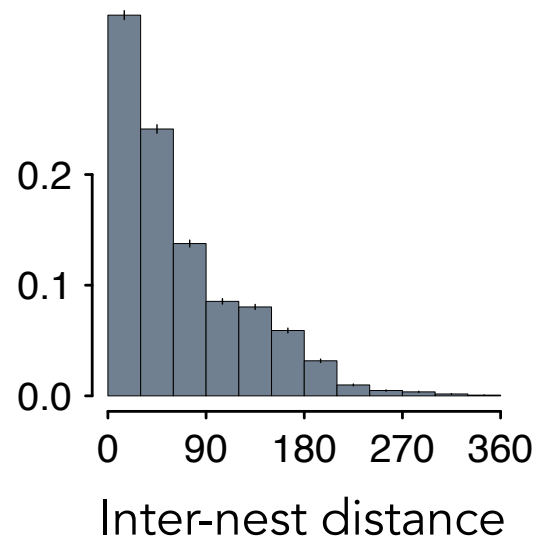

Nest location

Inter-nest distance

Supplement: Supplementary file 7 — Supplementary Material 7. [file 40850_2024_205_MOESM7_ESM.pdf]
