# Supplementary material for: Null models confirm nest site fidelity by male smallmouth bass, Micropterus dolomieu
Source: BMC Zool. 2024 Jun 27;9:13. doi: 10.1186/s40850-024-00205-z (PMC11210175; doi:10.1186/s40850-024-00205-z)
Supplement: Supplementary file 8 — Supplementary Material 8. [file 40850_2024_205_MOESM8_ESM.docx]

**Table S1.** Number of nests by category (coarse substrate or other; recently used or new) in which females spawned and occupancy rates by repeat breeders, 2002-2009. Margin totals are in bold.

|  |  | Occupancy | | | | | | |
| --- | --- | --- | --- | --- | --- | --- | --- | --- |
|  |  | Total | | |  | Repeat breeders | | |
| Year |  | Recent | New |  |  | Recent | New |  |
| 2002 | Coarse | 92 | 90 | **182** |  | 47 | 18 | **65** |
|  | Other | 20 | 39 | **59** |  | 9 | 5 | **14** |
|  |  | **112** | **129** |  |  | **56** | **23** |  |
|  |  |  |  |  |  |  |  |  |
| 2003 | Coarse | 114 | 98 | **212** |  | 76 | 42 | **118** |
|  | Other | 31 | 25 | **56** |  | 20 | 4 | **24** |
|  |  | **145** | **123** |  |  | **96** | **44** |  |
|  |  |  |  |  |  |  |  |  |
| 2004 | Coarse | 145 | 109 | **254** |  | 86 | 36 | **122** |
|  | Other | 21 | 54 | **75** |  | 9 | 15 | **24** |
|  |  | **166** | **163** |  |  | **95** | **51** |  |
|  |  |  |  |  |  |  |  |  |
| 2005 | Coarse | 116 | 51 | **167** |  | 85 | 32 | **117** |
|  | Other | 31 | 44 | **75** |  | 17 | 25 | **44** |
|  |  | **147** | **95** |  |  | **102** | **57** |  |
|  |  |  |  |  |  |  |  |  |
| 2006 | Coarse | 99 | 54 | **153** |  | 69 | 21 | **90** |
|  | Other | 31 | 30 | **61** |  | 22 | 15 | **37** |
|  |  | **130** | **84** |  |  | **91** | **36** |  |
|  |  |  |  |  |  |  |  |  |
| 2007 | Coarse | 57 | 65 | **122** |  | 41 | 40 | **81** |
|  | Other | 17 | 31 | **48** |  | 17 | 20 | **37** |
|  |  | **74** | **96** |  |  | **58** | **60** |  |
|  |  |  |  |  |  |  |  |  |
| 2008 | Coarse | 82 | 75 | **157** |  | 63 | 29 | **92** |
|  | Other | 18 | 36 | **54** |  | 10 | 6 | **16** |
|  |  | **100** | **111** |  |  | **73** | **35** |  |
|  |  |  |  |  |  |  |  |  |
| 2009 | Coarse | 127 | 41 | **168** |  | 85 | 17 | **102** |
|  | Other | 24 | 23 | **47** |  | 18 | 13 | **31** |
|  |  | **151** | **64** |  |  | **103** | **30** |  |
