# Supplementary material for: Null models confirm nest site fidelity by male smallmouth bass, Micropterus dolomieu
Source: BMC Zool. 2024 Jun 27;9:13. doi: 10.1186/s40850-024-00205-z (PMC11210175; doi:10.1186/s40850-024-00205-z)
Supplement: Supplementary file 9 — Supplementary Material 9. [file 40850_2024_205_MOESM9_ESM.docx]

**Table S2.** Proportions of coarse substrate nests (CSN) and recently used nests (RUN) occupied by repeat breeders and simulated proportions under the base null model. The observed (*O*) values of statistics not in an extreme tail of the distribution of simulated statistics are indicated in bold. Expected (*E*) values are based on 1,500 simulations under the basic null model. Ω is the frequency of the 1,500 simulated proportions that deviated more in absolute value from *E* than the difference between *E* and *O*.

|  | CSN | | |  | RUN | | |
| --- | --- | --- | --- | --- | --- | --- | --- |
| Year | *O* | *E* | Ω |  | *O* | *E* | Ω*** |
| 2002 | 0.36 | **0.33** | 0.1073 |  | 0.50 | 0.33 | < 0.0007 |
| 2003 | 0.56 | **0.53** | 0.0960 |  | 0.66 | 0.53 | < 0.0007 |
| 2004 | 0.48 | 0.44 | 0.0187 |  | 0.57 | 0.44 | < 0.0007 |
| 2005 | 0.70 | 0.61 | 0.0367 |  | 0.69 | **0.61** | 0.1480 |
| 2006 | 0.59 | **0.59** | 0.8820 |  | 0.70 | 0.59 | < 0.0007 |
| 2007 | 0.66 | **0.68** | 0.4487 |  | 0.76 | **0.68** | 0.0713 |
| 2008 | 0.59 | 0.51 | 0.0007 |  | 0.73 | 0.51 | < 0.0007 |
| 2009 | 0.61 | **0.62** | 0.6060 |  | 0.68 | 0.62 | 0.0047 |

* Ω < 0.0007 indicates that the observed proportion was not contained in the distribution of proportions generated in 1,500 simulations.
