# Supplementary material for: Null models confirm nest site fidelity by male smallmouth bass, Micropterus dolomieu
Source: BMC Zool. 2024 Jun 27;9:13. doi: 10.1186/s40850-024-00205-z (PMC11210175; doi:10.1186/s40850-024-00205-z)
Supplement: Supplementary file 10 — Supplementary Material 10. [file 40850_2024_205_MOESM10_ESM.docx]

**Table S3.** Expected proportions (*E*) of coarse substrate nests (CSN) and recently used nests (RUN) occupied by repeat breeders when null models include a preference for either CSN or RUN. Expected proportions are means based on 1,500 simulations under four nest access constraints (Bias: 1, 2, 5, 10).* Bolded *E* are the means of simulated distributions in which the observed proportion was contained.**

|  |  | Preference | | | | | | | | | | |
| --- | --- | --- | --- | --- | --- | --- | --- | --- | --- | --- | --- | --- |
|  |  | CSN | | | | |  | RUN | | | | |
|  |  | CSN | |  | RUN | |  | CS | |  | RUN | |
| Year | Bias | *E* | Ω |  | *E* | Ω |  | *E* | Ω |  | *E* | Ω |
| 2002 | 1 | **0.34** | 0.4073 |  | 0.33 | < 0.0007 |  | **0.32** | 0.1233 |  | 0.34 | < 0.0007 |
|  | 2 | 0.40 | 0.0020 |  | 0.35 | < 0.0007 |  | **0.34** | 0.2553 |  | 0.45 | 0.1293 |
|  | 5 | 0.43 | < 0.0007 |  | 0.36 | < 0.0007 |  | **0.35** | 0.5747 |  | **0.58** | 0.0033 |
|  | 10 | 0.43 | < 0.0007 |  | 0.36 | < 0.0007 |  | **0.35** | 0.8207 |  | 0.66 | < 0.0007 |
|  |  |  |  |  |  |  |  |  |  |  |  |  |
| 2003 | 1 | 0.59 | 0.0020 |  | 0.53 | < 0.0007 |  | 0.53 | 0.0953 |  | 0.59 | 0.0033 |
|  | 2 | 0.64 | < 0.0007 |  | 0.53 | < 0.0007 |  | **0.53** | 0.0727 |  | **0.68** | 0.4047 |
|  | 5 | 0.67 | < 0.0007 |  | 0.53 | < 0.0007 |  | 0.53 | 0.0227 |  | 0.79 | < 0.0007 |
|  | 10 | 0.67 | < 0.0007 |  | 0.53 | < 0.0007 |  | 0.53 | 0.0107 |  | 0.85 | < 0.0007 |
|  |  |  |  |  |  |  |  |  |  |  |  |  |
| 2004 | 1 | 0.50 | 0.0447 |  | 0.47 | < 0.0007 |  | 0.45 | 0.0500 |  | 0.50 | 0.0133 |
|  | 2 | 0.55 | < 0.0007 |  | 0.49 | < 0.0007 |  | **0.47** | 0.3480 |  | **0.61** | 0.1373 |
|  | 5 | 0.57 | < 0.0007 |  | 0.50 | 0.0047 |  | **0.48** | 1.0000 |  | 0.73 | < 0.0007 |
|  | 10 | 0.57 | < 0.0007 |  | 0.50 | 0.0040 |  | **0.49** | 0.3213 |  | 0.80 | < 0.0007 |
|  |  |  |  |  |  |  |  |  |  |  |  |  |
| 2005 | 1 | 0.66 | 0.0440 |  | **0.66** | 0.1793 |  | 0.66 | 0.0460 |  | **0.66** | 0.1620 |
|  | 2 | **0.72** | 0.4027 |  | **0.68** | 0.5833 |  | **0.67** | 0.1860 |  | **0.73** | 0.2120 |
|  | 5 | 0.79 | < 0.0007 |  | **0.70** | 0.8753 |  | **0.69** | 0.6360 |  | 0.80 | < 0.0007 |
|  | 10 | 0.83 | < 0.0007 |  | **0.71** | 0.4047 |  | **0.70** | 1.0000 |  | 0.84 | < 0.0007 |
|  |  |  |  |  |  |  |  |  |  |  |  |  |
| 2006 | 1 | **0.60** | 0.2540 |  | 0.59 | 0.0007 |  | **0.59** | 0.8827 |  | 0.60 | 0.0007 |
|  | 2 | 0.67 | < 0.0007 |  | 0.61 | < 0.0007 |  | **0.60** | 0.5253 |  | **0.68** | 0.4780 |
|  | 5 | 0.74 | < 0.0007 |  | 0.62 | 0.0013 |  | **0.61** | 0.2913 |  | 0.77 | 0.0120 |
|  | 10 | 0.79 | < 0.0007 |  | 0.62 | 0.0007 |  | **0.61** | 0.1393 |  | 0.81 | < 0.0007 |
|  |  |  |  |  |  |  |  |  |  |  |  |  |
| 2007 | 1 | **0.69** | 0.2540 |  | **0.68** | 0.0940 |  | **0.68** | 0.4647 |  | **0.69** | 0.1293 |
|  | 2 | 0.75 | < 0.0007 |  | **0.70** | 0.1767 |  | **0.69** | 0.3587 |  | **0.77** | 0.7353 |
|  | 5 | 0.82 | < 0.0007 |  | **0.71** | 0.1847 |  | **0.69** | 0.2500 |  | 0.85 | 0.0053 |
|  | 10 | 0.86 | < 0.0007 |  | **0.72** | 0.2740 |  | **0.69** | 0.1727 |  | 0.89 | < 0.0007 |
|  |  |  |  |  |  |  |  |  |  |  |  |  |
| 2008 | 1 | 0.52 | 0.0020 |  | 0.52 | < 0.0007 |  | 0.51 | < 0.0007 |  | 0.52 | < 0.0007 |
|  | 2 | **0.59** | 0.7127 |  | 0.54 | < 0.0007 |  | 0.52 | 0.0027 |  | 0.63 | 0.0040 |
|  | 5 | 0.67 | < 0.0007 |  | 0.56 | < 0.0007 |  | 0.53 | 0.0027 |  | **0.76** | 0.3327 |
|  | 10 | 0.69 | < 0.0007 |  | 0.56 | < 0.0007 |  | 0.54 | 0.0107 |  | 0.83 | < 0.0007 |
|  |  |  |  |  |  |  |  |  |  |  |  |  |
| 2009 | 1 | 0.69 | < 0.0007 |  | 0.64 | 0.0333 |  | **0.63** | 0.1720 |  | **0.69** | 0.7627 |
|  | 2 | 0.73 | < 0.0007 |  | **0.65** | 0.0820 |  | 0.64 | 0.0527 |  | 0.74 | 0.0020 |
|  | 5 | 0.77 | < 0.0007 |  | **0.66** | 0.2353 |  | 0.65 | 0.0007 |  | 0.80 | < 0.0007 |
|  | 10 | 0.79 | < 0.0007 |  | **0.67** | 0.2593 |  | 0.66 | < 0.0007 |  | 0.84 | < 0.0007 |

*Bias: 1, 2, 5 and 10 indicate that repeat breeders were equally, twice, five or ten times as likely to be assigned to a nest as a new breeder.

** The observed proportions for each year are found in Table S2. Ω < 0.0007 indicates that the observed proportion was not contained in the distribution of proportions generated in 1,500 simulations.
