# Supplementary material for: Null models confirm nest site fidelity by male smallmouth bass, Micropterus dolomieu
Source: BMC Zool. 2024 Jun 27;9:13. doi: 10.1186/s40850-024-00205-z (PMC11210175; doi:10.1186/s40850-024-00205-z)
Supplement: Supplementary file 11 — Supplementary Material 11. [file 40850_2024_205_MOESM11_ESM.docx]

**Table S4.** Expected inter-nest distance statistics based on 1,500 simulations in which a preference for coarse substrate nests was imposed. Bolded values of variances indicate that the observed variance of distances was contained in the distribution of the 1,500 simulated variances (Ω > 0.05).* *Bias* is the nest access advantage given to repeat breeders, where one indicates that repeat and new breeders had equal access to nests.** *Proportion* indicates the frequency of repeat breeders expected to occupy the same exact location in consecutive years.

|  |  | Statistics | | | | | |
| --- | --- | --- | --- | --- | --- | --- | --- |
| Episodes | Bias | Mean | Median | Variance*** | Skew | Kurtosis | Proportion |
| 2001-2002 | 1 | 585 | 630 | **89,260** | -0.27 | 1.92 | 0.0009 |
|  | 2 | 582 | 624 | **88,823** | -0.27 | 1.93 | 0.0009 |
|  | 5 | 579 | 622 | **88,774** | -0.27 | 1.92 | 0.0010 |
|  | 10 | 580 | 623 | **88,755** | -0.27 | 1.92 | 0.0010 |
|  |  |  |  |  |  |  |  |
| 2002-2003 | 1 | 582 | 622 | 91,375 | -0.23 | 1.87 | 0.0004 |
|  | 2 | 580 | 618 | 91,430 | -0.23 | 1.86 | 0.0003 |
|  | 5 | 578 | 616 | 91,372 | -0.22 | 1.86 | 0.0004 |
|  | 10 | 579 | 615 | 91,386 | -0.23 | 1.86 | 0.0004 |
|  |  |  |  |  |  |  |  |
| 2003-2004 | 1 | 570 | 607 | **86419** | -0.21 | 1.91 | 0.0003 |
|  | 2 | 567 | 601 | **85987** | -0.20 | 1.92 | 0.0003 |
|  | 5 | 565 | 598 | **86089** | -0.20 | 1.91 | 0.0004 |
|  | 10 | 564 | 597 | **86283** | -0.20 | 1.91 | 0.0004 |
|  |  |  |  |  |  |  |  |
| 2004-2005 | 1 | 582 | 630 | **86338** | -0.27 | 1.96 | 0.0003 |
|  | 2 | 583 | 632 | **85869** | -0.28 | 1.97 | 0.0003 |
|  | 5 | 582 | 629 | **85101** | -0.28 | 1.97 | 0.0003 |
|  | 10 | 581 | 628 | **85123** | -0.28 | 1.97 | 0.0003 |
|  |  |  |  |  |  |  |  |
| 2005-2006 | 1 | 590 | 640 | 86891 | -0.31 | 2.00 | 0.0009 |
|  | 2 | 588 | 639 | 85979 | -0.30 | 1.99 | 0.0009 |
|  | 5 | 585 | 633 | 85448 | -0.30 | 1.99 | 0.0010 |
|  | 10 | 583 | 630 | 85079 | -0.30 | 1.98 | 0.0010 |
|  |  |  |  |  |  |  |  |
| 2006-2007 | 0 | 584 | 630 | 85172 | -0.28 | 2.00 | 0.0005 |
|  | 2 | 579 | 624 | 84358 | -0.27 | 1.99 | 0.0005 |
|  | 5 | 577 | 622 | 83616 | -0.27 | 1.98 | 0.0003 |
|  | 10 | 576 | 618 | 83190 | -0.26 | 1.98 | 0.0004 |
|  |  |  |  |  |  |  |  |
| 2007-2008 | 1 | 561 | 600 | 84127 | -0.22 | 1.93 | 0.0014 |
|  | 2 | 558 | 593 | 83784 | -0.21 | 1.91 | 0.0017 |
|  | 5 | 556 | 589 | 83526 | -0.21 | 1.90 | 0.0015 |
|  | 10 | 555 | 587 | 83238 | -0.21 | 1.90 | 0.0017 |
|  |  |  |  |  |  |  |  |

**Table S4** (continued)

|  |  | Statistics | | | | | | |
| --- | --- | --- | --- | --- | --- | --- | --- | --- |
| Episodes | Bias | Mean | Median | Variance*** | Skew | Kurtosis | | Proportion |
| 2008-2009 | 1 | 560 | 600 | 83134 | -0.26 | 1.90 | | 0.0017 |
|  | 2 | 558 | 598 | 83332 | -0.25 | 1.89 | | 0.0016 |
|  | 5 | 555 | 595 | 83386 | -0.25 | 1.88 | | 0.0017 |
|  | 10 | 554 | 592 | 83460 | -0.24 |  | 1.87 | 0.0017 |

* The observed values of statistics are found in Table 2 of the primary paper.

** Please see Appendix 2 for a precise definition of the nest access advantage to repeat breeders.

*** The observed values of all distance statistics, except the variance, were not contained in any of the 1,500 simulated distributions generated for each access bias (Ω < 0.0007). The row-wise Ω for bolded variances are 0.4800, 0.4967, 0.5067, 0.5307 (2001-2002); 0.4733, 0.5233, 0.4847, 0.4647 (2003-2004); and 0.1973, 0.2080, 0.2533, 0.2720 (2004-2005).
