# Supplementary material for: Null models confirm nest site fidelity by male smallmouth bass, Micropterus dolomieu
Source: BMC Zool. 2024 Jun 27;9:13. doi: 10.1186/s40850-024-00205-z (PMC11210175; doi:10.1186/s40850-024-00205-z)
Supplement: Supplementary file 12 — Supplementary Material 12. [file 40850_2024_205_MOESM12_ESM.docx]

**Table S5.** Expected inter-nest distance statistics based on 1,500 simulations in which a preference for recently used nests was imposed. Bolded values of variances indicate that the observed variance was contained in the distribution of the 1,500 simulated variances (Ω > 0.05).* *Bias* is the nest access advantage given to repeat breeders, where one indicates that repeat and new breeders had equal access to nests.** *Proportion* indicates the frequency of repeat breeders expected to occupy the same exact location in consecutive years.

|  |  | Statistics | | | | | |
| --- | --- | --- | --- | --- | --- | --- | --- |
| Episodes | Bias | Mean | Median | Variance*** | Skew | Kurtosis | Proportion |
| 2001-2002 | 1 | 586 | 631 | **89418** | -0.27 | 1.92 | 0.0008 |
|  | 2 | 583 | 627 | **89850** | -0.27 | 1.92 | 0.0010 |
|  | 5 | 582 | 628 | **89189** | -0.28 | 1.92 | 0.0014 |
|  | 10 | 580 | 627 | **89043** | -0.28 | 1.93 | 0.0016 |
|  |  |  |  |  |  |  |  |
| 2002-2003 | 1 | 582 | 621 | 91710 | -0.23 | 1.87 | 0.0004 |
|  | 2 | 580 | 619 | 91627 | -0.22 | 1.86 | 0.0005 |
|  | 5 | 579 | 617 | 92213 | -0.22 | 1.85 | 0.0006 |
|  | 10 | 578 | 615 | 92248 | -0.22 | 1.85 | 0.0006 |
|  |  |  |  |  |  |  |  |
| 2003-2004 | 1 | 575 | 614 | **87524** | -0.22 | 1.92 | 0.0004 |
|  | 2 | 573 | 611 | **88260** | -0.21 | 1.90 | 0.0004 |
|  | 5 | 571 | 608 | **89266** | -0.20 | 1.88 | 0.0005 |
|  | 10 | 570 | 606 | **89718** | -0.20 | 1.87 | 0.0006 |
|  |  |  |  |  |  |  |  |
| 2004-2005 | 1 | 583 | 632 | **86291** | -0.28 | 1.97 | 0.0003 |
|  | 2 | 582 | 631 | **86449** | -0.28 | 1.96 | 0.0003 |
|  | 5 | 581 | 628 | **86389** | -0.27 | 1.95 | 0.0004 |
|  | 10 | 580 | 628 | **86129** | -0.27 | 1.95 | 0.0003 |
|  |  |  |  |  |  |  |  |
| 2005-2006 | 1 | 591 | 642 | 87018 | -0.31 | 1.99 | 0.0009 |
|  | 2 | 592 | 642 | 87181 | -0.31 | 1.99 | 0.0010 |
|  | 5 | 593 | 643 | 87387 | -0.31 | 1.99 | 0.0011 |
|  | 10 | 592 | 641 | 87566 | -0.30 | 1.98 | 0.0013 |
|  |  |  |  |  |  |  |  |
| 2006-2007 | 0 | 583 | 629 | 85158 | -0.28 | 1.99 | 0.0005 |
|  | 2 | 584 | 631 | 85256 | -0.28 | 2.00 | 0.0006 |
|  | 5 | 584 | 630 | 86222 | -0.28 | 1.98 | 0.0007 |
|  | 10 | 584 | 631 | 86175 | -0.28 | 1.99 | 0.0007 |
|  |  |  |  |  |  |  |  |
| 2007-2008 | 1 | 561 | 598 | 84444 | -0.22 | 1.93 | 0.0016 |
|  | 2 | 559 | 593 | 84255 | -0.21 | 1.92 | 0.0019 |
|  | 5 | 555 | 589 | 84509 | -0.19 | 1.91 | 0.0022 |
|  | 10 | 552 | 583 | 84873 | -0.18 | 1.90 | 0.0025 |
|  |  |  |  |  |  |  |  |
|  |  |  |  |  |  |  |  |

**Table S5** (continued)

|  |  | Statistics | | | | | | |
| --- | --- | --- | --- | --- | --- | --- | --- | --- |
| Episodes | Bias | Mean | Median | Variance*** | Skew | Kurtosis | | Proportion |
|  |  |  |  |  |  |  | |  |
| 2008-2009 | 1 | 561 | 602 | 83371 | -0.25 | 1.91 | | 0.0018 |
|  | 2 | 561 | 602 | 83400 | -0.26 | 1.91 | | 0.0020 |
|  | 5 | 559 | 600 | 83868 | -0.25 | 1.90 | | 0.0021 |
|  | 10 | 560 | 601 | 83697 | -0.25 |  | 1.91 | 0.0024 |

* The observed values of statistics are found in Table 2 of the primary paper.

** Please see Appendix 2 for a precise definition of the nest access advantage to repeat breeders.

*** The observed values of all distance statistics, except the variance, were not contained in any of the 1,500 simulated distributions generated for each access bias (Ω < 0.0007). The row-wise Ω for bolded variances are 0.4860, 0.4633, 0.4947, 0.5033 (2001-2002); 0.3820, 0.3373, 0.2253, 0.2153 (2003-2004); and 0.2080, 0.1807, 0.1793, 0.2023 (2004-2005).
